# A new AI assisted approach aligns data standards and accelerates interoperability in biomedical research

**DOI:** 10.1038/s41746-026-02795-z

**Published:** 2026-06-12

**Authors:** Rodney Alan Long, Shannon Ballard, Syed Shah, Owen Bianchi, Lietsel Jones, Mathew J. Koretsky, Nicole Kuznetsov, Elise Marsan, Bryant Jen, Philip Chiang, Abhradeep Mukherjee, Cornelis Blauwendraat, Hampton Leonard, Dan Vitale, Kristin Levine, Sara Bandres-Ciga, Paige Jarreau, Patrick Brannelly, Mukta Phatak, Caroline Pantazis, Laurel Screven, Kate Andersh, Alifiya Kapasi, John F. Crary, David Gutman, Brittany N. Dugger, Sarah Biber, Timothy Hohman, Faraz Faghri, Michael Griswold, Lana Sargent, Kendall van Keuren-Jensen, Andrew B. Singleton, Yang Fann, Mike A. Nalls, Hirotaka Iwaki

**Affiliations:** 1 Center for Alzheimer’s and Related Dementias, National Institute on Aging, National Institute of Neurological Disorders and Stroke, National Institutes of Health, Bethesda, MD USA; 2 DataTecnica LLC, Washington, DC, USA; 3 Office of Intramural Research, Office of the Director, National Institutes of Health, Bethesda, MD USA; 4 Coalition for Aligning Sciences, Washington, DC, USA; 5 10,000 Brains Project., Oakland, CA USA; 6 Rush University Medical Center, Chicago, IL USA; 7 Departments of Pathology, Neuroscience, and Artificial Intelligence & Human Health, Neuropathology Brain Bank & Research CoRE, Ronald M. Loeb Center for Alzheimer’s Disease, Friedman Brain Institute, Icahn School of Medicine at Mount Sinai, New York, NY USA; 8 Emory University, Atlanta, GA USA; 9 University of California - Davis, Sacramento, CA USA; 10 University of Washington, Seattle, WA USA; 11 Vanderbilt University, Nashville, TN USA; 12 The MIND Center, Jackson, MS USA; 13 Virginia Commonwealth University, Richmond, VA USA

**Keywords:** Software, Classification and taxonomy, Computational platforms and environments, Data integration, Data processing, Software, Standards

## Abstract

We demonstrate how Large Language Models (LLMs) accelerate biomedical data harmonization through automated Common Data Element (CDE) generation. We processed 31 datasets including clinical taxonomies and research data dictionaries through OpenAI’s Generative Pre-trained Transformer - 4 (API Model gpt-4-0613), generating comprehensive metadata for each element using a template-based system. Subject-matter experts validated outputs, finding 94% of generated metadata fields required no revision overall, with an unweighted accuracy of 83.8%, unweighted, for semi-structured sources. Dramatically faster than manual approaches. Our system uses ElasticSearch with weighted field matching to identify semantic equivalences between variables, avoiding duplicate CDEs while building a standardized repository. Testing with Alzheimer’s Disease Neuroimaging Initiative (ADNI) and Global Parkinson’s Genetic Program (GP2) datasets showed 32.4% of previously unseen headers successfully mapped to our CDEs, with interoperability scores averaging 53.8/100 based on matching, completeness, and compliance metrics. This approach automates the most tedious aspects of data integration, reducing barriers to cross-study collaboration in biomedical research.

## Introduction

Artificial intelligence/Machine learning (AI/ML) provides rapidly evolving tools for data processing and harmonization, offering particular value for biomedical research areas like Alzheimer’s disease and related dementias (ADRD), where data harmonization can unlock new insights^1,2^. Federal health agencies, including the National Institutes of Health (NIH), now prioritize “AI-readiness,” with a focus on establishing methods for enhanced data discovery and interoperability across large organizations^3,4^.

While formal data standards are developed through consensus processes by standards organizations, our work provides an AI-assisted methodology to accelerate the implementation and harmonization of these established standards across diverse datasets. In our experience, leveraging Large Language Models (LLMs) accelerates the data wrangling process by an order of magnitude, automating tedious harmonization tasks that would otherwise require manual effort. This automation is particularly valuable in healthcare, where data harmonization and defining common elements traditionally demand labor-intensive processes from researchers and practitioners^5–8^.

Collaborative data science increasingly relies on the principles of Findability, Accessibility, Interoperability, and Reusability, known collectively as FAIR. These principles address the well-known need to effectively combine data from different health information systems^9^. At the heart of achieving this integration are Common Data Elements (CDEs), standardized definitions and metadata that help researchers clearly interpret and connect data across multiple studies and sources. Traditional approaches to CDE implementation primarily rely on lexical matching, manual curation, and rule-based mapping to standardize data elements from pre-existing, structured datasets^5,10^. While effective for specific domains with standardized input data, these methods are inherently limited by the need for extensive manual intervention and typically cover only a small subset of domains^5,11,12^. By contrast, our AI-assisted approach significantly reduces manual effort and extends to non-standardized datasets, allowing researchers to focus more effectively on scientific investigation rather than data wrangling^6,9,13^.

In this paper, we present an AI-assisted methodology for generating Common Data Elements (GenCDEs) and harmonizing biomedical datasets. This methodology has been implemented to support the FAIRkit platform (Findable, Accessible, Interoperable, Reusable toolkit), which serves as a foundation for enhanced data discovery in biomedical research. Our approach combines AI-driven CDE generation with human expert oversight to rapidly standardize data elements across diverse sources. As a case study of this methodology’s application, we focus on data elements within the ADRD realm alongside heterogeneous clinical data from 72 cataloged studies. The methodology enabled us to generate and audit over 42,000 GenCDEs across 31 of these studies, effectively reducing the data wrangling required for cross-study interoperability within the FAIRkit platform.

Our work builds upon existing medical terminologies and classification systems, including Systematized Nomenclature of Medicine (SNOMED), International Classification of Diseases (ICD), Healthcare Common Procedure Terminology System (HCPCS), National Institutes of Health (NIH) Common Data Element Repository, and Common Data Elements (CDEs) from various research initiatives. Rather than creating another siloed standard, we accelerate the identification and alignment of established standards. This approach significantly reduces the effort needed for cross-study interoperability. Our HITL (Human-In-The-Loop) methodology pairs subject-matter experts with AI tools, applying open science practices to make data silos interoperable faster.

Automating the process of assessing data interoperability is key to AI-readiness in the biomedical space, particularly in light of recent developments relating to data locality requirements and federated learning. With data discovery and data wrangling consuming a large portion of data science efforts, our approach aims to decrease the activation energy for researchers and research networks, freeing up time for more innovative scientific inquiry. This framework will not only provide a growing collection of reference biomedical CDEs, but also create a dynamic repository that becomes progressively more effective at harmonizing disparate datasets. As more datasets are processed, the system learns to recognize increasingly nuanced semantic relationships between data elements, effectively expanding its capability to bridge different research domains and accelerate collaborative science.

## Results

### AI-accelerated generation of experimental and clinical CDEs

Table 1 summarizes the effectiveness of using applied-AI to accelerate the creation of CDEs. This process automates much of the tedious foundational work of looking up variable names, aliases and permissible values as well as formatting data outputs. This efficiency saves the experts in the HITL system immense amounts of time compared to de-novo construction.

**Table 1:** Sources and accuracy of generated CDEs

Origin	*n* Elements	Hit rate (%)	Source category
Biomedical Translational Research Informatics (BTRIS) Clinical Observations	12,437	100	Established Coding System
Center for Alzheimer’s and Related Dementias (CARD)	621	87	Sparse Dictionary
Clinical Trials Database (CTDB) Form Legends	1578	87	Sparse Dictionaries/Just Headers
Current Procedural Terminology - Healthcare Common Procedure Coding System (CPT – HCPCS)	596	100	Established Coding System
Gastrointestinal Symptom Rating Scale (GSRS)	15	60	Sparse Dictionary
Health Rhythms and Inferences	99	91	Sparse Dictionary
ICD	16,989	100	Established Coding System
Input Exercise Protocol	6	100	Sparse Dictionary
Intake Form Protocol	282	33	Sparse Dictionary
Kubio Heart Rate Variability (HRV)	27	80	Sparse Dictionary
Metabolic Cart	38	69	Sparse Dictionary
MNPQ Protocol: A keystroke assessment that measures typing speed and average intervals	14	64	Sparse Dictionary
Montreal Cognitive Assessment (MoCA)	15	93	Sparse Dictionary
National Institute of Mental Health (NIMH) Life Chart	147	78	Sparse Dictionary
National Library of Medicine (NLM) Endorsed	137	100	Established Coding System
National Library of Medicine (NLM) Qualified	6308	83	Established Coding System
Neuropsychiatric	1105	80	Sparse Dictionary
Ninehole Assessment	7	94	Sparse Dictionary
Non-Motor Symptom assessment scale for Parkinson’s Disease (NMSS)	100	73	Sparse Dictionary
Nutrition Data System for Research (NDSR)	120	100	Sparse Dictionary
Populations Underrepresented in Mental Illness Association Studies (PUMAS)	1143	60	Just Headers
Pronutra	12	100	Sparse Dictionary
PsyToolKit	10	100	Sparse Dictionary
Resting State Electroencephalogram (EEG)	101	100	Sparse Dictionary
Rush Alzheimer’s Disease Center (RADC)	79	90	Sparse Dictionary
Safety Monitor Survey	27	100	Sparse Dictionary
Short Form Survey - 36 (SF-36)	36	50	Sparse Dictionary
Symp PsyTool Kit	26	100	Sparse Dictionary
Timed Up and Go (TUG)	8	96	Sparse Dictionary
Unified Parkinson’s Disease Rating Scale (UPDRS)	125	97	Sparse Dictionary
MIMICEL (MIMIC-IV Event Log for Emergency Department)	171	98	Manual Inputs by Staff
	Total	Weighted Score	
	42,379	94	

This table describes the 31 data sources used for CDE generation. ‘Origin’ refers to the input data source, including general ICD-10 coding from the NIH Clinical Center. ‘N elements’ represents the number of unique data objects that could potentially be converted into CDEs. ‘Hit rate’ indicates the percentage of metadata fields per CDE that did not require manual editing by the expert reviewer. ‘Source category’ specifies the data structure type used to build the CDEs. For web links to ingested content, please see the Supplementary Information. A sparse dictionary may reflect a lack of extended titles (compared to abbreviated headers), standardization in data formatting and entry, and/or descriptions.

The AI-driven approach significantly accelerated the generation of CDEs from data headers, medical coding systems, and sparse or established data dictionaries. Across all data sources, GPT-4 (OpenAI’s Generative Pre-trained Transformer - 4) achieved a weighted accuracy rate of 94.0% in generating CDEs, with accuracy defined as the percentage of generated fields that required no manual revisions and weighted by averaging across CDE metadata fields rather than across data sources.

84.6% (35,853) of CDEs were derived from established clinical classification systems, while 15.4% (6,508) were generated from semi-structured sources such as data headers and sparse data dictionaries. The overall unweighted accuracy, which averaged accuracy by source rather than per element, was 85.9%. When excluding established clinical classification systems, the unweighted hit rate for CDEs generated from semi-structured sources was 83.8%. This semi-structured hit rate represents performance in a more challenging real-world environment.

These results demonstrate the efficacy of AI models in automating CDE generation, particularly when dealing with structured clinical systems. Variability in hit rates across data sources suggests that further optimization may be needed for semi-structured or sparse data inputs.

### ElasticSearch matching of data fields to CDEs

Headers from sources external to the material used to construct the current set of CDEs successfully matched in 25.8% of cases. These headers were pulled from the Alzheimer’s Disease Neuroimaging Initiative (ADNI) and the Global Parkinson’s Genetics Program (GP2), our two test cohorts^14,15^. We also tested against clinical inputs including manually entered free-text notes from MIMIC-IV Event Log (MIMICEL) from the NIH Clinical Center^16–19^.

At the time of writing, our CDE repository contained a subset of clinically relevant ICD codes. A total of 13,425 unique ICD codes from the MIMICEL dataset were correctly matched at a rate of 48.9%. From the data set, 27,660 unique terms in clinician notes were extracted from recorded chief complaints, of which 4.4% were matched to existing CDEs within our repository. This is summarized in Table 2.

**Table 2:** Mapping test results

Table	Term source	*n* Terms	Confirmed match (%)
ADNI Merge	Column header	116	12.1
ADNI MOCA	Column header	56	17.8
ADNI Adverse Symptoms (ADSX)	Column header	41	34.1
GP2	Column header	421	29.4
MIMICEL Diagnosis	ICD column	13,425	48.9
MIMICEL Triage	Chief complaint column	27,660	4.4

Description of the six tables used to test matching capabilities. Data table refers to a specific table from a set. Term source indicates whether terms were extracted from data headers or a specific column. ‘N terms’ indicates how many unique terms were extracted from the table. This includes data headers, ICD codes, and the documented chief complaints of a patient. The ‘Confirmed match (%)’ is the percentage of returned matches that were verified to be correct.

### Assessing interoperability for data silos

We applied our interoperability scoring framework to test datasets from the Alzheimer’s Disease Neuroimaging Initiative (ADNI) and the Global Parkinson’s Genetics Program (GP2), which quantified the current state of compatibility with our CDE repository and highlighted specific challenges in achieving off-the-shelf harmonization (Table 3).

**Table 3:** Interoperability test results

Table	*n* columns	Matching (%)	Completeness (%)	Compliance (%)	Interoperability score
GP2	421	29.4	16.4	94.6	46.8
ADNI Merge	116	12.1	53.8	100	55.3
ADNI MoCA	56	17.8	90.8	75	61.2
ADNI Alzheimer’s Diagnosis and Symptom Checklist	41	34.1	100	21.4	51.8

Interoperability results based on three tables. ‘N columns’ indicate the total number of columns for the table. Matching reports the percentage of columns that were accurately matched to a CDE within our repository. Completeness reports the percentage of non-null fields for a matched column. Compliance is the percentage of non-null values that were within the bounds of our defined permissible value set. Interoperability score is the average score of the three criteria.

While these data sets have established dictionaries, they were not ingested into our CDE repository prior to testing in order to assess real-world mapping performance and interoperability potential. Prior ingestion would have artificially inflated both matching rates and interoperability scores through perfect string matches, undermining our evaluation of how the system handles semantically similar variables across independently developed datasets.

A sparse GP2 dataset from multiple clinical sites was used for testing. Of 421 data headers, 29.4% were confirmed as correct matches, 16.4% of the matched data fields contained non-missing values, and 94.6% of those values were recognized and in compliance with the permissible value set. This suggests that data that can be matched and is complete is generally within tolerances and nearly analysis ready. Although, sparsity led to a total interoperability score of 46.8%.

ADNI Merge is a table that represents the consolidation of multiple ADNI research phases. Of the 116 data headers, 12.1% were correctly matched to CDEs in our repository. Of those matches, 53.8% of the fields were populated and 100% of the values adhered to the set permissible values. An interoperability score of 55.3% was achieved. ADNI MoCA contains data related to the Montreal Cognitive Assessment. Of the 56 column headers, 17.8% were matched to existing CDEs within the repository. Of the matched data headers, 90.8% of the fields were populated and 75% of them complied with set permissible values. The overall interoperability score was calculated to be 61.2%. ADNI ADSXLIST refers to the Alzheimer’s Diagnosis and Symptom Checklist. Of the 41 data headers, 34.1% matched established CDEs within our repository. Matched columns were 100% populated and 21.4% of the values complied with set permissible values. The calculated interoperability score for this table was 51.8%.

These estimates across ADNI sub-studies illustrate how different aspects of the data harmonization process and the inherent difficulty of harmonization even within studies can be quantified by the interoperability scoring system described here. Low matching rates to existing CDEs is the primary problem for automated harmonization and off-the-shelf interoperability. As this system grows, this should improve.

### Validation of CDE generation consistency and reliability

**Fig. 1:**
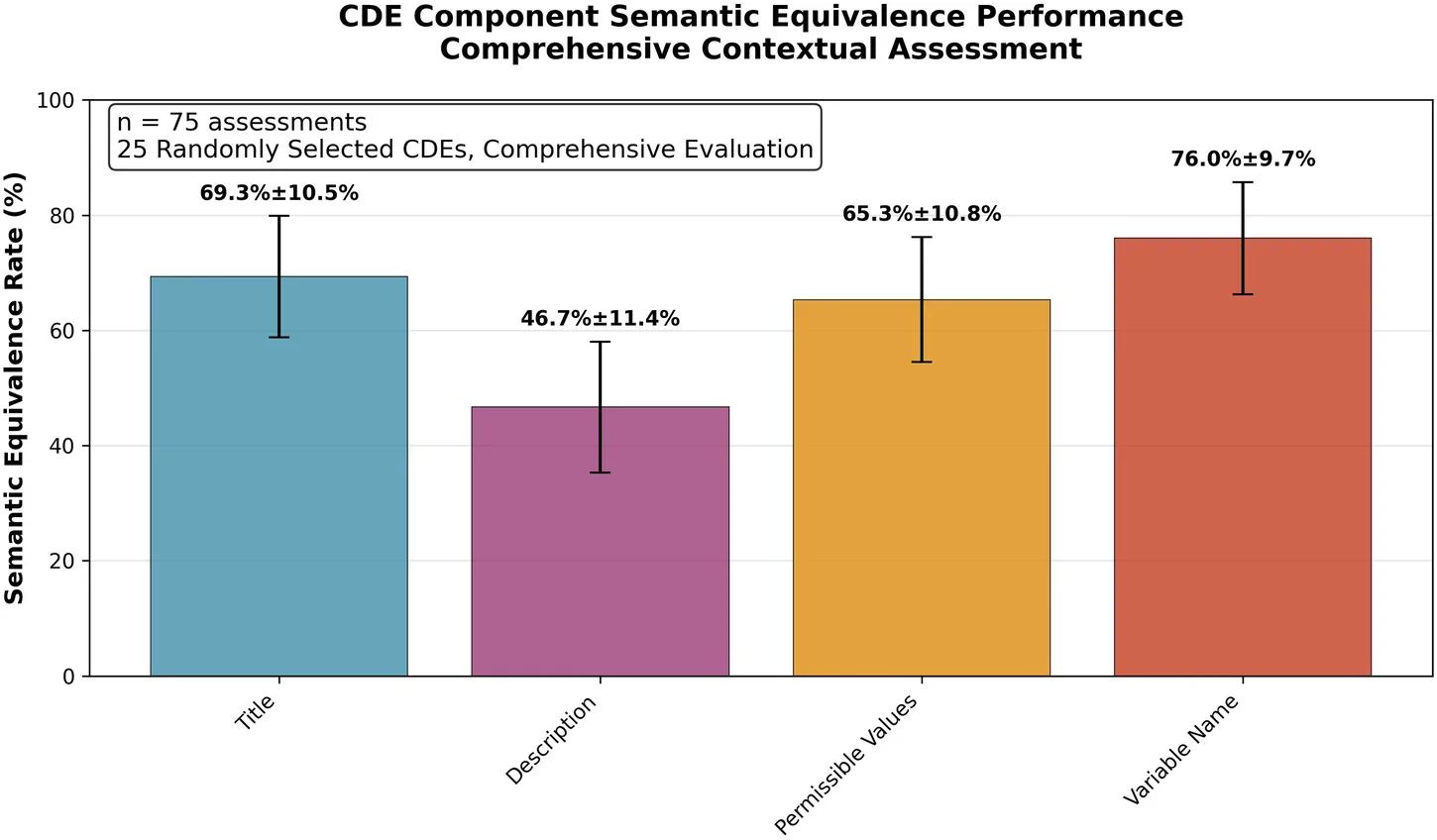
CDE component semantic equivalence performance. Bar chart showing semantic equivalence rates with standard errors for four CDE metadata components (title, description, permissible values, and variable name) based on comprehensive contextual assessment of 25 randomly selected CDEs across multiple generation runs. Variable names demonstrate the highest equivalence at 76.0% ± 9.7%, followed by titles at 69.3% ± 10.5% and permissible values at 65.3% ± 10.8%, while descriptions show greater variability at 46.7% ± 11.4%.

Our statistical validation demonstrated robust consistency in AI-assisted CDE generation across multiple independent runs. Analysis of 25 randomly selected CDEs revealed high semantic equivalence rates for critical metadata components, with variable names achieving 76.0% ± 9.7% equivalence and titles showing 69.3% ± 10.5% equivalence across runs (Fig. 1). Permissible values demonstrated 65.3% ± 10.8% equivalence, while descriptions showed greater variability at 46.7% ± 11.4% equivalence.

**Fig. 2:**
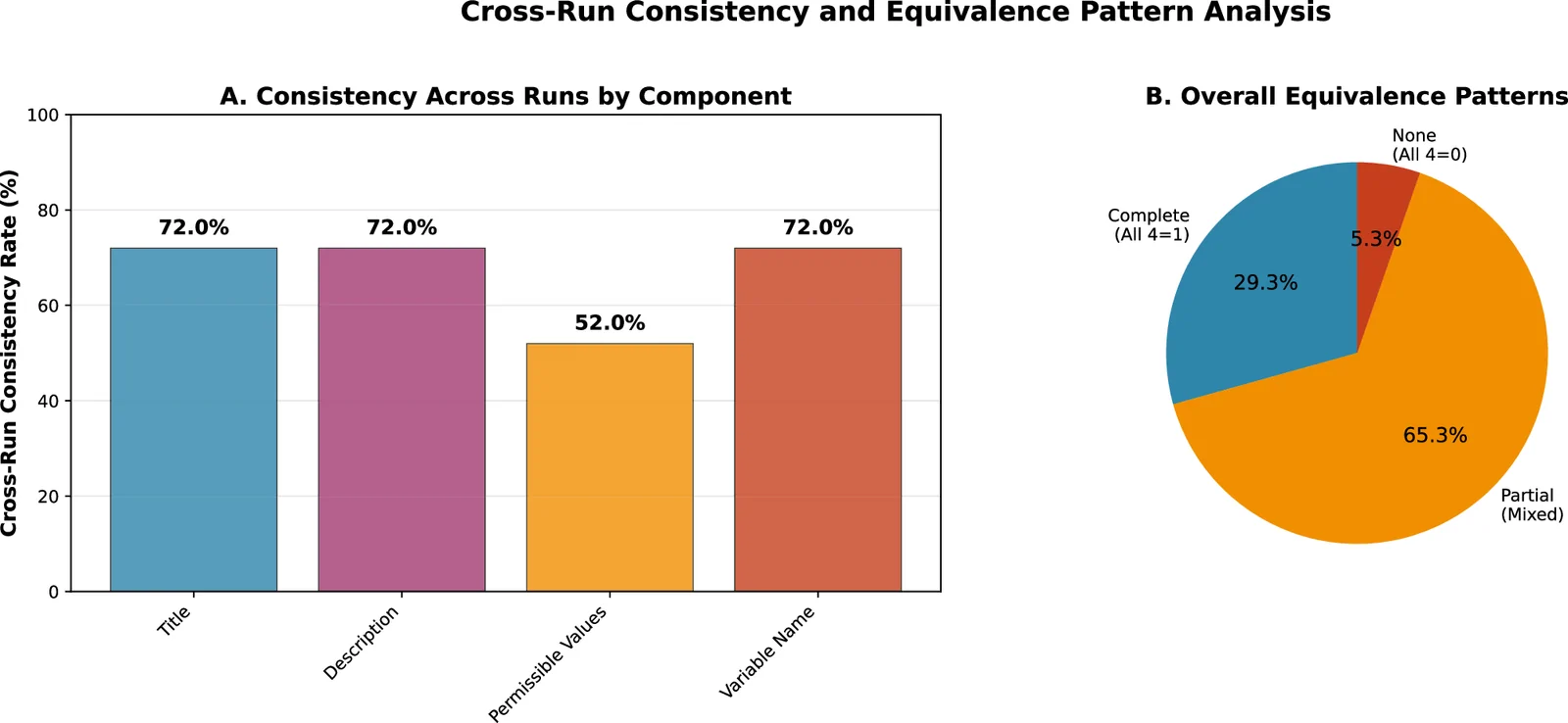
Cross-run consistency and equivalence pattern analysis. **A** Bar chart showing consistency rates between Run 2 and Run 3 for each CDE component, with titles, descriptions, and variable names each achieving 72% consistency, while permissible values show 52% consistency. **B** Pie chart illustrating the distribution of overall equivalence patterns: complete equivalence (29.3%), partial equivalence (65.3%), and no equivalence (5.3%).

**Fig. 3:**
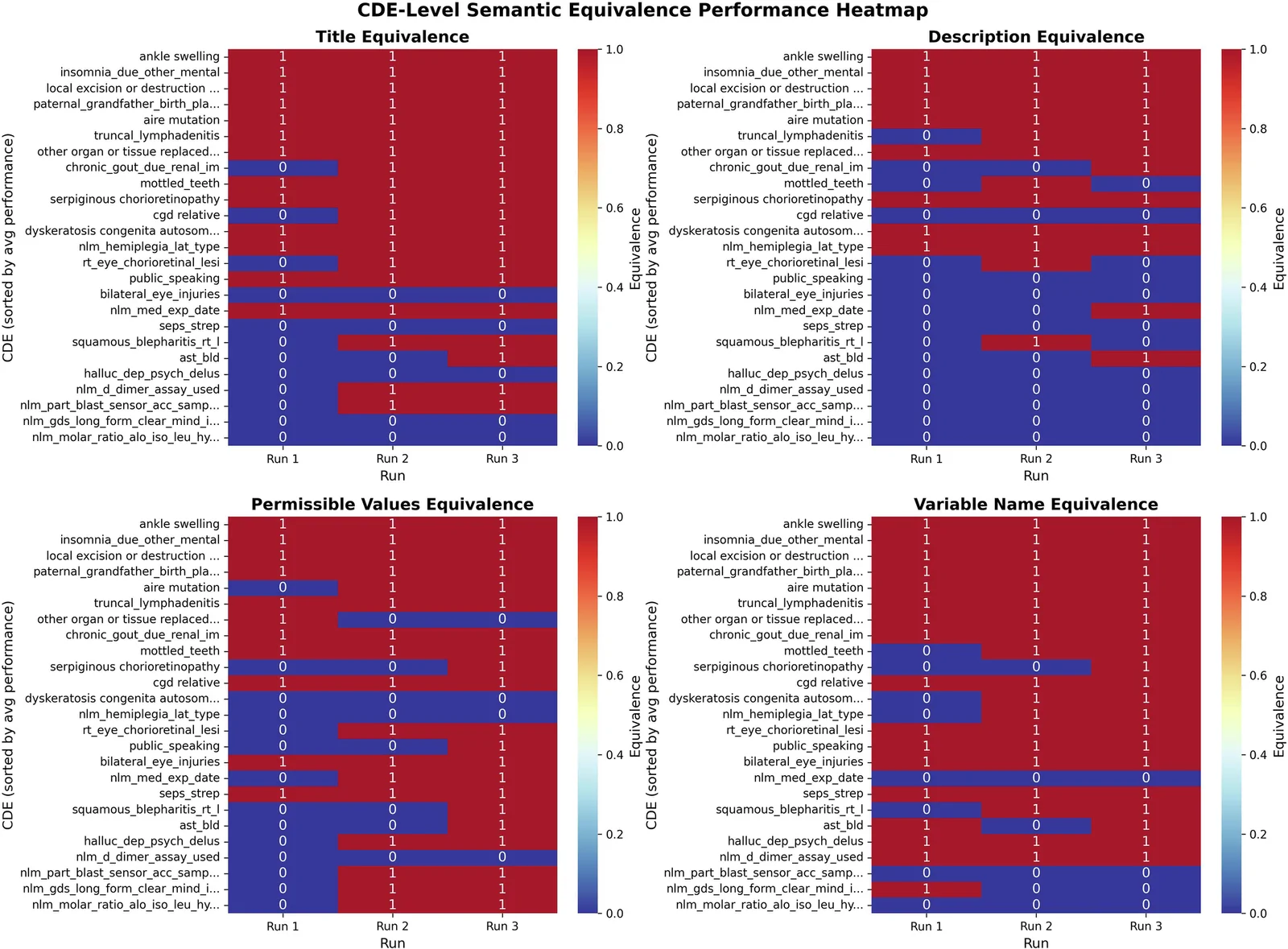
CDE-level semantic equivalence performance heatmap. Detailed heatmap showing individual CDE performance across all four metadata components for Run 2 and Run 3. CDEs are sorted by average performance, with darker red indicating semantic equivalence (1) and darker blue indicating non-equivalence (0). The visualization demonstrates that high-performing CDEs maintain consistency across runs, while variability is primarily concentrated in specific CDEs rather than being randomly distributed.

The analysis identified that 29.3% of CDEs achieved complete semantic equivalence across all four components, while 65.3% showed partial equivalence with variation in one or more components (Fig. 2B). Notably, only 5.3% of CDEs demonstrated complete non-equivalence across all components. Cross-run consistency analysis revealed strong reproducibility, with variable names, titles, and descriptions each achieving 72% consistency between runs, while permissible values showed 52% consistency (Fig. 2A), indicating the fundamental reliability of the generation process Fig. 3.

Component-specific analysis revealed differential performance patterns across CDE metadata fields. Variable names and titles, which represent the most structured and concise metadata elements, consistently achieved the highest equivalence rates. The lower equivalence rate for descriptions (46.7%) reflects the greater linguistic flexibility inherent in longer-form text generation, though this variability does not impact functional interoperability as descriptions serve primarily documentary rather than computational purposes.

These validation results demonstrate that despite the probabilistic nature of large language models, our methodology achieves reproducibility levels comparable to or exceeding traditional manual curation approaches, while maintaining the substantial efficiency gains of automation.

## Discussion

This work demonstrates the transformative impact of AI-assisted methodologies in advancing biomedical research, particularly in enhancing data interoperability and generating CDEs. While our interoperability scoring was tested on neurology-focused cohorts (ADNI and GP2) as a challenging real-world use case, it is important to note that the underlying CDE repository was generated from 31 highly diverse biomedical datasets. As shown in Table 1, these sources span general clinical taxonomies (ICD, CPT-HCPCS), nutrition (NDSR), gastroenterology (GSRS), and mental health (PUMAS), demonstrating the domain-agnostic nature of the CDE generation methodology itself. By combining current AI models, specifically OpenAI’s GPT-4, with expert human oversight, we have dramatically accelerated the generation of CDEs and streamlined data harmonization across diverse data silos. Our system automates many of the tedious tasks involved in, resulting in significant efficiency gains.

At peak efficiency, a single Subject-Matter Expert (SME) could audit over 4000 CDEs per week, an order of magnitude improvement over conventional methods that typically produce fewer than 400 CDEs weekly. For comparison, the NIH Helping to End Addiction Long-term (HEAL) Initiative’s Integrative Management of Chronic Pain and OUD Opioid Use Disorder) for Whole Recovery (IMPOWR) Network required ~3–4 months for a working group to select and define 800 CDEs^5^. Our manual auditing step was critical in refining the CDEs by identifying errors or inconsistencies requiring intervention. While efficiency varies by subject matter and source (averaging ~1500 CDEs per week), these results demonstrate how automating tedious harmonization tasks under expert oversight can accelerate biomedical data standardization.

These efficiency gains are directly linked to the accuracy of our AI-driven approach, which achieved a weighted accuracy rate of 94.0% in generating CDEs, significantly reducing the amount of manual work required and enabling the rapid generation of large quantities of CDEs, far faster than could be achieved through manual methods. This high accuracy, with 84.6% of CDEs derived from established clinical classification systems, demonstrates the method’s robust performance when applied to structured data sources. Structured data, characterized by well-defined variables and consistent formats, allows AI models to effectively generate and standardize CDEs that are uniformly formatted, leading to quicker and more reliable outcomes. However, this performance notably differs when dealing with semi-structured datasets, as explored below.

When the approach was applied to semi-structured data sources, such as sparse data dictionaries, the accuracy rates were slightly lower (83.8% unweighted and 90.8% weighted). Semi-structured data, which often needs more consistent formatting and can be more ambiguous, presents greater challenges for AI models. To illustrate these challenges concretely, we examine specific examples from datasets like NDSR and Intake Form Protocol.

An illustrative example of a sparse yet effective dataset is the Nutrition Data System for Research (NDSR) data dictionary. Notably, the acronym ‘NDSR’ itself is never explicitly defined within the dictionary. Moreover, the ‘title’ metadata field merely replicates variable names with spaces inserted, while the ‘short description’ field duplicates the ‘title’ without further elaboration. Definitions in the NDSR data dictionary provide minimal expansion beyond the titles, often appearing as fragmented text that fails to clearly convey semantic relationships between terms. Despite these limitations, advanced natural language processing models such as OpenAI’s GPT-4 can still extract meaning from these entries, accurately expanding the acronym ‘NDSR’ and generating appropriate metadata for each data element.

In contrast, the Intake Form data dictionary presents similar but more pronounced challenges. Many data elements lack standardization and are custom-defined, while definitions frequently mirror the title without adding meaningful explanation. As an example, GPT-4 struggled to interpret abbreviations like ‘Qpd’ (Questionnaire - Parkinson’s Disease) without explicit context linking the abbreviation to Parkinson’s disease, tremors, or established assessment tools designed specifically for Parkinson’s patient populations. These interpretive difficulties directly impact the audit performance of AI-generated CDEs, as highlighted below.

An audit comparing the quality of generated CDEs underscores these distinctions clearly. Specifically, the NDSR-derived CDEs achieved a 100% audit pass rate, in contrast to the Intake Form Protocol, which only attained a 33% audit success rate. The superior audit performance of NDSR-derived CDEs can be attributed to their incorporation of critical contextual information, such as specific time points for data collection (e.g., pre-screening, admission, post-discharge) and explicit references to macronutrients and measurement units. Conversely, the Intake Form Protocol predominantly employed abstract alphanumeric codes (e.g., A1, Gtxt, F2txt), significantly diminishing interpretability and thus resulting in lower audit outcomes. Addressing these shortcomings may require integrating more advanced technologies, such as ElasticSearch, as explored in the subsequent analysis.

Building upon the findings of the audit, integrating ElasticSearch into the CDE matching process underscores the critical role of advanced search technologies in biomedical data management. While the success rate in matching headers from external sources was moderate (25.8%), the ability to correctly map 48.9% of ICD codes from a large-scale dataset like MIMIC-IV demonstrates the robustness of this approach for clinical data. It is important to note that Table 1 reflects the repository’s content at the time of analysis, which contained a specific subset of ICD codes, rather than a limitation of the matching methodology itself. As the CDE repository expands, this match rate is expected to increase substantially. However, the lower matching rates observed for unstructured terms (4.4%) from recorded chief complaints suggests that enhancing the natural language processing capabilities of the system or providing additional contextual information could improve the accuracy and consistency of CDE generation from unstructured or semi-structured data sources. Future improvements in natural language processing or enhanced contextual inputs will likely be essential to improve accuracy and consistency in processing unstructured and semi-structured biomedical data.

These technical findings translate directly to practical applications with significant implications for biomedical research workflows. Our findings demonstrate significant efficiency gains in CDE generation, with peak production rates exceeding 4000 CDEs per week per subject-matter expert under optimal conditions. This peak efficiency came from processing structured sources, like ICD-10 diagnostic codes. Here, we used their existing titles which were great source material. For diagnostic CDEs that simply indicate the presence or absence of a condition, disease, or procedure, we assigned standardized binary permissible values (0|1 or True|False) rather than generating them through AI. The existing mappings between ICD-10 and SNOMED further accelerated the process by providing foundational relationships to established clinical taxonomies. These conditions created an ideal scenario for rapid CDE generation and verification, as there were fewer metadata fields to audit and those that were generated were of good quality.

Processing ambiguous sources with sparse dictionaries led to lower efficiency rates, around 1500 CDEs per week. However, our method still greatly exceeded traditional manual methods, which usually generate fewer around 400 CDEs per week with full-time work. As repositories grow, this efficiency gap becomes more significant. Manual methods encounter more challenges as the scale increases. As CDE repositories expand, finding synonymous CDEs that are duplicates gets tougher. A CDE repository of our size would make it nearly impossible for experts to keep track of all existing elements using manual and traditional methods. Simple string matching approaches prove insufficient due to their inability to capture semantic similarities, terminology variations, and conceptual relationships between data elements.

Our ElasticSearch implementation with weighted fields and fuzzy matching directly addresses these scaling limitations. We suggest that using AI in the context of Retrieval-Augmented Generation (RAG) could be a valid method for repository management^20^. The benefit of RAG comes from giving AI direct access to source and working material. In this case, creating a knowledge graph as the foundation for RAG appears promising, as it is well suited for mapping relationships between objects^21^. This method wasn’t as accessible as it is currently when we undertook this task.

This efficiency differential has direct implications for biomedical research initiatives:

In multi-center studies, speeding up data harmonization helps remove a substantial hurdle for collaboration between institutions. Research networks can set up common data models faster. This can cut down study startup times. As a result, resources can be redirected from data standardization and mapping work toward actual scientific inquiry and analysis.

A key practical outcome of this work is the interoperability scoring system, which provides a tangible metric for assessing dataset compatibility. This system moves beyond theoretical assessment by offering a direct, quantitative tool for research planning. By scoring datasets on matching, completeness, and compliance, our method allows investigators to prospectively estimate the manual effort required for harmonization, prioritize resources effectively, and avoid investing in datasets that are fundamentally incompatible. This directly addresses a common challenge in observational research, where data heterogeneity can delay projects and complicate meta-analyses.

The reproducibility of AI-assisted methods represents a fundamental challenge in computational approaches to data standardization. Our comprehensive statistical validation provides quantitative evidence that LLM-based CDE generation produces consistent outputs despite the inherent probabilistic nature of these models. The demonstrated semantic equivalence rates of 69–76% for structured components (titles, variable names, and permissible values), coupled with cross-run consistency rates of 52–72% across three independent runs, indicate that the methodology achieves reliability levels suitable for production use. These reproducibility metrics are particularly noteworthy given that traditional consensus-based CDE development processes, while deterministic in theory, often show substantial inter-rater variability in practice, with studies reporting agreement rates between domain experts ranging from 60 to 85% for metadata classification tasks.

The differential performance across CDE components offers important insights into the strengths and limitations of AI-assisted metadata generation. The strong consistency observed for titles, descriptions, and variable names (72% cross-run consistency each) indicates that current language models perform reliably for both structured and unstructured text generation tasks. The lower consistency for permissible values (52%) reflects the inherent complexity of generating structured value sets that must align with clinical standards and measurement constraints. This variability pattern aligns with where human expertise proves most valuable in the HITL process, validating and standardizing permissible value sets that require domain-specific knowledge. The systematic nature of these performance differences suggests that targeted improvements in prompt engineering or model fine-tuning for value set generation could further enhance overall consistency.

Our finding that only 5.3% of CDEs showed complete non-equivalence across all components provides strong evidence for the fundamental reliability of the generation process. The predominance of partial equivalence patterns (65.3% of cases) suggests that the model maintains conceptual consistency even when specific phrasings vary, a pattern that mirrors the natural variation observed in human-generated metadata. This level of consistency, achieved without fine-tuning or few-shot learning, demonstrates that general-purpose large language models have reached sufficient maturity for structured metadata generation tasks in biomedical research contexts.

The validation methodology employed here, utilizing comprehensive contextual assessment rather than simple string matching, provides a more accurate measure of functional equivalence than purely lexical comparisons. This approach recognizes that semantic equivalence in scientific metadata does not require identical text but rather consistent meaning and utility. The three-run validation design demonstrates that consistency patterns hold across multiple independent generation attempts, establishing that the observed performance reflects systematic model behavior rather than random chance. These findings suggest that the combination of LLM-based generation with human oversight represents a viable path forward for accelerating the development of comprehensive CDE repositories while maintaining quality standards necessary for scientific research.

These findings have important implications for the scalability of data harmonization efforts. The demonstrated consistency of our approach, combined with the order-of-magnitude efficiency gains reported in our original results, suggests that AI-assisted methods can accelerate the development of comprehensive CDE repositories without sacrificing quality. As these repositories grow and models continue to improve, we anticipate further enhancements in both consistency and efficiency, ultimately reducing barriers to data interoperability in biomedical research.

With the rapid advancement of GPT models, we have also seen the expansion of context windows and reduced token cost. Our approach utilized a zero-shot prompting, which means we did not provide specific examples, nor did we provide feedback or corrections. With recent developments, providing AI direct access to the CDE repository is a more feasible approach. As mentioned previously, the mapped CDEs could be converted to a knowledge graph, where nodes represent CDEs and edges describe the properties of each relationship. Theoretically, the AI model could generate graph queries to navigate the relationships to find an ideal match and potentially get us closer to fully automated mapping. Beyond that, it could possibly assist with full repository audits by locating redundant CDEs that may have gone undetected using our original mapping techniques, or even suggest CDEs to fill observed gaps in coverage.

Fine-tuning the AI model on domain-specific datasets could be a valuable next step to address these challenges. Fine-tuning allows the model to more precisely adjust to the specific context of the data it is processing, improving its ability to generate accurate CDEs from unstructured, semi-structured, or novel data sources. This process could reduce the need for manual oversight and increase the system’s efficiency, particularly in specialized fields where data may not conform to widely established standards.

The project also underscored the importance of effective data preprocessing. Enhancements in preprocessing techniques could mitigate some of the challenges associated with unstructured data. For instance, developing more sophisticated methods to contextualize ambiguous terms and applying organizing principles tailored to the specific characteristics of the data could further reduce the potential for misinterpretation by the model. These improvements would maintain the system’s high accuracy across a broader range of data types.

Another area for future improvement involves the human-in-the-loop process. While essential for ensuring the quality of the generated CDEs, the reliance on manual review introduces a bottleneck in scalability. Automating certain aspects of the quality assurance process, such as implementing machine learning-based checks to flag potential errors for human review, could streamline this step. This would enhance the system’s scalability, making it more suitable for larger or more complex datasets without compromising accuracy.

A limitation of our study is the absence of a direct quantitative comparison with other data harmonization platforms. A formal benchmark is challenging, as existing tools are often designed for different use cases and inputs. For instance, while OHDSI’s Usagi maps source codes via keyword-based TF-IDF, advanced deep learning models like TOKI(Trustworthiness Oracle through Keyword Identification) have since been developed to perform more accurate semantic classification between two well-defined code descriptions^22^. However, both of these are fundamentally mapping tools designed to translate existing, structured source vocabularies into a standard one. In contrast, our AI-assisted methodology addresses the preceding “first-mile” problem: generating rich, structured CDE metadata from sparse, ambiguous, and non-coded inputs like raw column headers. Furthermore, our ElasticSearch-based matching system is not a one-to-one classifier like TOKI, but an information retrieval engine designed for repository management, to search and map variables against a large, evolving library of over 42,000 CDEs. Given these differences in core purpose (metadata generation and repository-scale retrieval versus pairwise code translation), a direct benchmark is not straightforward. Future work should focus on establishing evaluation frameworks that can assess and compare these different but complementary approaches to data harmonization. Additionally, while our HITL process relied on collaborative review, we did not formally calculate inter-rater reliability (e.g., using Cohen’s kappa) to quantify the consistency of SME revisions; incorporating such a metric would be a valuable addition to future studies.

In conclusion, this project has demonstrated the significant potential of AI-driven solutions in the field of biomedical data standardization. The high accuracy rates achieved, particularly with structured data, highlight the system’s capability to reduce the manual labor involved in CDE generation, offering substantial improvements in both speed and efficiency. While there are areas for further refinement, particularly in handling semi-structured or novel data, the project’s overall success provides a strong foundation for automated CDE generation. By incorporating fine-tuning, enhancing preprocessing techniques, and improving scalability through automation, this system could become an even more powerful tool in standardizing biomedical data, ultimately advancing research efforts across diverse scientific domains.

## Methods

### Ethics statement

We exclusively used publicly accessible data without including any patient and/or participant-related or confidential information in AI applications. According to local regulations this is considered non-human subjects research and no ethics approval was required for this study. All procedures were conducted in accordance with the Declaration of Helsinki.

### Input data

To date, we have cataloged and indexed 72 datasets to enable FAIR metadata inventory and discovery, 31 of which contributed to the generation of subsequent Common Data Elements (CDEs) (see Supplementary Information). Metadata was manually cataloged for each study using Google Forms or the FAIRkit app to create a comprehensive internal inventory at NIH.

For CDE generation, we ingested 31 datasets containing primarily clinical data, supplemented by variables defined from diverse sources, including SNOMED, ICD, HCPCS, the NIH Common Data Element Repository, and various research initiative-based and epidemiological data dictionaries.

In cases where comprehensive data dictionaries or metadata files were absent, we utilized column headers directly from datasets. We identified sparse data dictionaries as those characterized by limited metadata fields, infrequent population of fields, duplicated elements without informative context, and undefined abbreviations requiring extensive cross-referencing with original study materials for clarification. Detailed examples of these issues and the resolution process are elaborated in the Methods subsection ‘Human-in-the-Loop’ and the Discussion.

Dataset column names requiring additional clarification were identified and processed using OpenAI’s GPT-4 model. This approach allowed the translation of abbreviated or specialized terminology into comprehensive, standardized descriptions suitable for cross-study analyses.

After initial cataloging, we selected a subset of six datasets containing participant-level data for a proof-of-concept analysis, matching newly harmonized CDEs to existing datasets. This process allowed us to calculate interoperability scores across data silos, as detailed later in this document. No participant-level data was shared with AI models or external services; analysts exclusively determined concept matches and verified dataset compliance with permissible CDE values.

**Fig. 4:**
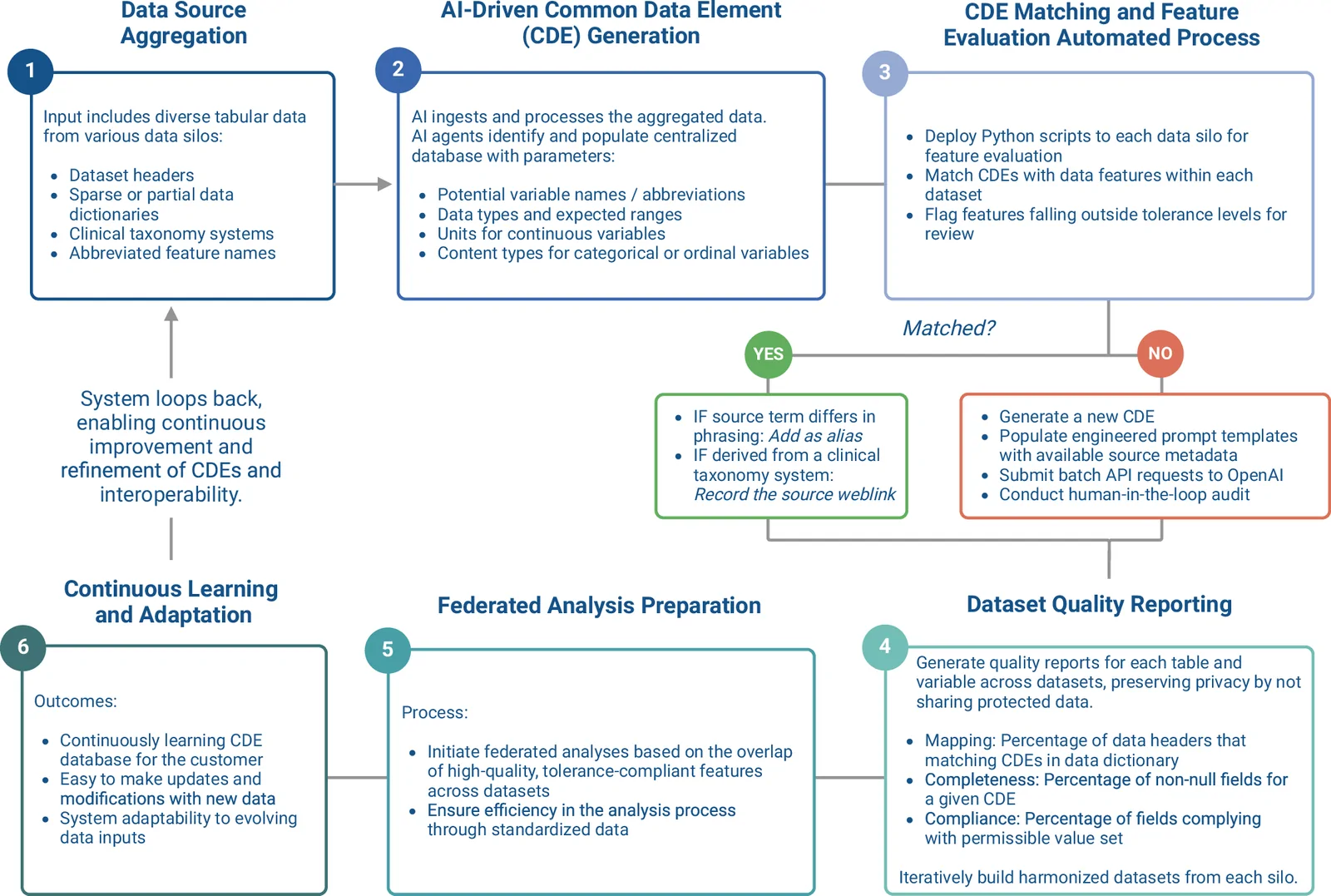
Workflow used to generate CDEs for interoperability assessments. This Figure illustrates the comprehensive workflow used to generate Common Data Elements (CDEs) for interoperability assessments. The process begins with diverse input data sources, including column headers, sparse and partial dictionaries, and clinical taxonomy systems. These inputs are processed through a GPT model with iterative prompt templates to generate structured CDE metadata such as titles, descriptions, and permissible values. Subject-matter experts provide oversight in a human-in-the-loop approach, reviewing AI-generated outputs for accuracy and relevance. The system incorporates ElasticSearch to match new entries against existing CDEs, avoiding duplication and enhancing searchability. The resulting CDEs are stored in a centralized repository, enabling automated interoperability assessment across previously siloed datasets. This framework significantly accelerates the traditionally labor-intensive process of data harmonization in biomedical research. Created in BioRender. Jarreau, P. (2025)^31^ https://BioRender.com/r61p979.

Please refer to Fig. 4 for a summary of the input data and the human-in-the-loop CDE system in general.

### AI-assisted CDE generation

We employed an AI-assisted approach to generate CDEs using column headers and data dictionaries from the 31 datasets ingested into FAIRkit without including any individual data points. We leveraged iterative prompt engineering and template-based automation. Abbreviated data headers and other elements requiring definition were extracted from diverse data sources, see Table 1. These elements were processed through fourth generation OpenAI GPT models (GPT-4 API endpoint model gpt-4-0613), accessed in a structured workflow to iteratively generate metadata elements, including alternative titles, abbreviations, permissible values, units of measure, preferred question text, and short descriptions. The complete analysis cost less than $1000 USD.

Our CDE generation pipeline processes three types of inputs: column headers from research datasets, sparse dictionaries, and clinical taxonomy systems. Existing metafields are extracted as text strings. These text strings are inserted into prompt templates when deemed contextually relevant to the metadata we intend to generate. For example, if permissible values were available within a sparse data dictionary, they can be useful to the AI as it attempts to extrapolate the meaning of a variable name (used as column headers in a dataset) when generating a descriptive element title. This newly generated data element title efficiently captures the core semantic meaning of the element and enhances AI’s ability to generate text related to what the data element signifies.

We then attempt to match them with existing CDEs in our repository using ElasticSearch. Elements that cannot be mapped undergo a structured generation process using the Jinja templating engine, which allows for systematic automation of prompts sent to OpenAI’s GPT models. For each element requiring generation, we employ a sequence of templates to produce complete CDE definitions. Initially, a template receives the raw column header or element name and produces an expanded, descriptive title. This title is then inserted into subsequent templates to generate additional metadata fields such as short descriptions, question text, data types, and permissible values. For example, when processing an abbreviated header like “NDSR1water6Txt,” the first template prompts the GPT model to expand it into a comprehensive title, which then serves as input for generating appropriate question text and permissible values.

This templating approach ensures consistency while allowing for customization based on the specific characteristics of each input source. Subject-matter experts review the generated content throughout this process, providing additional context when necessary for ambiguous elements, thereby maintaining quality through a human-in-the-loop methodology. The entire workflow is designed to reduce the manual effort traditionally required for cross-study data harmonization, while ensuring the resulting CDEs meet rigorous standards for clarity and interoperability.

Tokens are the metric by which LLM computation is measured and billed for. OpenAI states that a word is worth 1.25 tokens on average. Tokens can also be consumed by sub words and trailing spaces. We set the token parameter to cap responses at 100 tokens. The token limit was more than generous and was set as a consideration for collecting and generating a list of aliases that a CDE could potentially refer to among disparate datasets. On average, only 13 tokens were required to complete a response.

The temperature parameter controls the randomness or creativity of the AI model’s output^23^. It typically ranges from 0 to 1 (or sometimes higher), with different effects on the model’s response generation. The model balances predictability and creativity. Lower temperatures (e.g., 0.2–0.5) produce more focused and precise outputs, while higher temperatures (e.g. 0.7–1.0) generate more diverse and potentially more creative responses. For our purposes, the temperature parameter was set between 0.5 (data formats) and 0.7 (generating a list of aliases).

Custom prompt engineering was performed by inserting generated metadata fields using the Jinja templating engine^24^ for the automation and standardization of prompts sent to the AI models. For instance, if the CDE source was an abbreviated data header, such as ‘wbc_count’, it was inserted into the prompt template instructing the GPT model to expand it into a full, descriptive data element title. This expanded title was inserted into subsequent prompt templates for further metadata generation. The next step typically involved generating a short description of the data element, followed by creating question text to retrieve the data of interest. Once the data title and short description were available, they were inserted into a template to classify the data element by type (e.g., numerical continuous, categorical ordinal, or binary).

For numerical data types, the generated data title, type, and question text were inserted into a template that tasked the GPT model with assigning a unit of measure, where applicable. The unit of measure and question text were then used to generate a set of permissible values denoting acceptable content and ranges per CDE.

### Prompting templates for CDE generation

Our CDE generation process utilized a set of custom Jinja templates, each designed for specific metadata fields. These included templates for expanding abbreviated headers into descriptive titles, generating concise clinical explanations, creating standardized assessment questions, determining appropriate data formats, establishing permissible value sets, setting clinically appropriate boundaries, and generating alternative terms from medical ontologies. Each template incorporated contextual sections for domain-specific knowledge and related column data, particularly valuable for sparse dictionaries.

The content within each Jinja template was structured to solicit precisely the information needed for each metadata field. Templates began with clear, task-specific instructions (e.g., “Convert the following variable name into a clear, descriptive title by expanding abbreviations”) followed by a dedicated section presenting the input column within delimiter markers. This structure ensured the model focused on the exact term requiring expansion or description. Critical to our design were the contextual enhancement sections, where additional information could be conditionally included using Jinja’s conditional syntax ({% if usr_added_prompt_txt %} blocks). The prompt templates include two distinct context sections: one to provide domain-specific knowledge or study purpose through user text input, and another where relevant columns from the source data dictionary can be included. This contextual information is particularly valuable for sparse dictionaries or datasets with non-standard abbreviations, where successful interpretation often depends on understanding the broader research context rather than the isolated header alone (please refer to methods subsection Human-in-the-Loop for an example). This allowed incorporation of domain context and related column values when available, reducing the impact of information gaps on output quality.

For value-specific templates like permissible values, the prompts included explicit output format instructions tailored to different data types. Each format was illustrated. For instance, ‘List: Value1, Value2, Value3’ for categorical variables, ‘Range: (Value1-Value2) for continuous numerical data, and ‘Binary: 0, 1’ for boolean fields. These format-specific examples served as structural guides, ensuring the AI model would generate properly formatted outputs matching the underlying data type requirements. The templates were deliberately designed to constrain responses to the required information by specifying exact output formats and directing the model to only provide the requested value without extraneous text, facilitating consistent downstream processing across all generated CDEs. Prompt templates are included in the Supplementary Information.

### Human-in-the-loop

Throughout the generation of Common Data Elements (CDEs), a human-in-the-loop (HITL) approach played a crucial role in auditing AI-generated outputs. Typically, the process began with the generation of a descriptive title for each CDE from abbreviated headers. Subject-matter experts (SMEs), which includes two clinical data scientists (RAL, LJ), a medical doctor (HI), and two biomarker scientists (KvJ, EM), who assessed the accuracy of each AI-generated title to ensure correct interpretation of source data and sufficient detail for subsequent metadata generation. They performed ad-hoc revisions. Modifying prompt sequences or including additional metadata based on source complexity and quality was also left to their discretion.

Our HITL approach is essential for addressing inherent LLM limitations. While the probabilistic nature of generative models poses a challenge to achieving perfect computational reproducibility, our templated workflow provides a methodological consistency often absent in traditional, consensus-based CDE development. To mitigate the risk of biases inherited from the model, such as *vocabulary frequency bias*, where common terms may be favored over precise ones, or *anchoring bias* where experts may be unduly influenced by the initial AI suggestion, our process relies on a multi-disciplinary team of SMEs who collaboratively review and validate all outputs. This expert oversight, including consultation with additional specialists for ambiguous terms, serves as the primary safeguard against these potential issues.

For CDEs generated from abbreviated headers or sparse dictionaries, SMEs cross-referenced the AI-generated interpretations with the original source contexts when available. When ambiguity was detected, particularly in sparse dictionaries with lower hit rates, we implemented additional context-gathering steps before accepting AI-generated content. This included verification against established medical references and ontologies where applicable, and consultation with additional domain experts when necessary. The higher manual revision rates observed in some sparse dictionaries (as shown in Table 1) reflect this rigorous approach to distinguishing between merely plausible AI outputs and factually accurate interpretations.

As an example, the following header was encountered: *IQ_CS_SCD_HR_EX*. We consider this to be a complex header as you have five significantly abbreviated items using somewhat ubiquitous letter combinations. The quality could be considered poor due to the terms being severely abbreviated and commonly applied to other concepts. Initially, GPT interpreted it as *Interviewee’s Clinical Survey Weeknight Exercise Hours*. The correct expansion in this case was *Intake Questionnaire - Child Survey - School Day - Hours of Exercise*. In such situations, it is important to add additional context so that GPT may correctly interpret the abbreviations. For this example, it was important to include that the table contained data from an intake questionnaire for school children to assess and compare the hours of physical activity on school days vs weekends. In this case, the context stated that the column headers were sourced from a study that utilized an intake questionnaire. The questionnaire was designed to measure the hours of physical activity in school children. Specifically, to perform a comparative analysis of school days to weekends. This resulted in the correct expansion.

This hybrid approach, the combination of AI-driven metadata generation with expert oversight, helped ensure that the resulting CDEs were both accurate and contextually relevant. Once generated and reviewed, the CDEs were integrated into the centralized CDE repository with an updated and extended version of our CDEs hosted on HuggingFace [Reference of Parameters (RoP) CDEs, https://huggingface.co/datasets/DataTecnica/RoP_biomedical] which serves as a large-scale repository for standardized elements. This AI-assisted process significantly accelerated the creation of CDEs, reducing the time required to establish data harmonization across studies. For more on this, please see the results’ subsection *Time Savings for Novel CDE Generation*.

The auditing process involved scoring each CDE based on the percentage of metadata fields needing manual revision. The criteria for manual revision were based on specific, identifiable errors in the AI-generated output, which included: 1) Factual Incorrectness, such as an inaccurate expansion of a source abbreviation; 2) Internal Inconsistency, where generated metadata fields were not logically aligned, for instance, a mismatch between the question text and permissible value types; and 3) Contextual Misalignment, where the AI’s interpretation, while plausible, was incorrect for the specific source dataset. A common issue with ambiguous terms that required SMEs to provide additional context for the AI to interpret them correctly. Minor corrections, such as removing extraneous prefixes like “Permissible Values:” automatically added by GPT, were efficiently addressed via bulk updates or post-processing scripts and were not counted among manual edits. Although a JSON response mode was available, it was unreliable during data collection, as noted by users in the OpenAI community forum. The recent introduction of structured output by OpenAI (August 6, 2024) may improve future applications^25^. For numerical data elements, permissible ranges were derived from explicit definitions in source materials, observed data ranges, established clinical standards, or appropriate context based on units of measure. For categorical and ordinal data, including severity scales, permissible values defined in the original source were preserved if subject-matter experts (SMEs) deemed them broadly applicable, such as widely recognized clinical assessments or established disease progression scales. Conversely, when SMEs encountered unique, undefined, or narrowly defined scales specific to individual studies, these scales were not preserved as separate standards. Instead, they were mapped as aliases to existing CDEs, resulting in lower compliance scores during interoperability assessments (see Interoperability Scoring).

When source materials did not provide explicit permissible values and no suitable existing CDE was available for mapping, our framework utilized large language models (LLMs) to propose permissible values. These LLM-generated suggestions leveraged extensive biomedical research literature, benefiting from the LLM’s training on a corpus larger than any single research team could feasibly review manually. Subject-matter experts (SMEs) then rigorously reviewed these suggestions, verifying them against original documentation, established clinical references, and their domain expertise to ensure scientific accuracy and practical utility.

### Learning system and ElasticSearch

To expand our Common Data Elements (CDEs) repository, we applied a systematic approach that combined search technologies with expert curation across various datasets. This process involved three key phases: repository index creation, query processing, and subject-matter expert validation. ElasticSearch was employed to enhance the efficiency of matching existing data fields with appropriate CDEs. By leveraging ElasticSearch’s powerful text-based search capabilities, we were able to compare field names, descriptions, and associated metadata with a pre-existing repository of CDEs.

For the repository index creation phase, we established a CDE repository in ElasticSearch with a two-shard, one-replica setup to balance performance and redundancy. Custom analyzers were developed to accurately tokenize varying case formats such as snake_case and CamelCase, ensuring consistent processing of terminology variations. Each CDE in our repository was indexed with multiple metadata fields, including titles, aliases, short descriptions, preferred question text, and units of measure. These fields collectively provided the semantic foundation for matching operations. To learn more about the technical aspects of index creation utilizing ElasticSearch, please refer to the cited documentation^26,27^.

In the query processing phase, our team extracted column headers, variable names, and relevant metadata from source datasets as search queries against the indexed repository. For each source dataset, these elements were processed through ElasticSearch using a hybrid search methodology that combined traditional keyword-based retrieval with semantic vector representations. Semantic vectors (or embeddings) convert text into numerical representations that capture semantic meaning, allowing the system to detect conceptual similarities even when terminology differs, a critical capability given the terminological variations across research domains^28^.

To optimize matching accuracy, we implemented a weighted field strategy that prioritized the most informative metadata fields based on empirical analysis of their discriminative power. Aliases and title fields received the highest weight (4) because they typically contain the most concentrated semantic information and distinctive terminology. Short descriptions received the next highest weight (3) as they provide expanded context but with more variable phrasing. Preferred question text (2) and unit of measure (1) received lower weights as they contribute supplementary rather than primary identifying information. This weighting hierarchy was established after analyzing the information density and uniqueness of each field type across our repository.

The Best Matching 25 (BM25) algorithm was used to calculate relevance scores between query elements and indexed CDEs. This algorithm determines relevance based on term frequency while factoring in document length to reduce bias toward larger text fields, a critical consideration when matching concise column headers against more extensive CDE descriptions. We employed default BM25 parameters (k1 = 1.2, b = 0.75) with fuzziness set to ‘auto’, which adjusts matching tolerance based on term length (allowing for minor spelling variations and abbreviation differences).

To integrate results from the keyword-based (BM25) search and semantic vector-based retrieval, we applied a manual Reciprocal Rank Fusion (RRF) method^29^. The RRF method combined the ranked lists from both search methods into a single, unified ranking by calculating a cumulative relevance score based on each document’s position in the respective ranked lists. Higher cumulative RRF scores indicated stronger overall semantic correspondence.

In the validation phase, subject-matter experts reviewed the three highest-scoring matches for each source element. These experts evaluated semantic equivalence and contextual appropriateness, to determine whether a true match existed or if the element required creation of a new CDE. When source elements matched existing CDEs in meaning but differed in terminology, they were recorded as aliases. This approach balanced computational efficiency with domain expertise, addressing the challenges of terminology variability across research domains while maintaining semantic integrity in the harmonization process.

We consider this method to be a learning system as each time a new dataset is processed, the system not only maps variables to existing CDEs but also captures new terminology variations as aliases, effectively expanding its semantic understanding. These aliases become part of the repository’s knowledge base, improving future matching operations by recognizing a wider range of domain-specific terms for the same underlying concepts. Unlike static mapping systems, our framework becomes progressively more effective at identifying equivalences between seemingly disparate variables as it processes more datasets.

This progressive enrichment reduces the manual curation burden over time, as previously unrecognized terminology patterns become part of the system’s matching capabilities. The continuous feedback loop between computational matching and expert validation ensures that the system’s learning remains scientifically accurate while becoming increasingly autonomous in identifying semantic equivalences across diverse research contexts.

Our technology selection balanced functionality requirements with practical implementation considerations. For search functionality, we selected ElasticSearch due to its open-source availability, flexible text analysis capabilities, and configurable ranking algorithms. While we explored alternative approaches including similarity queries using Pinecone vector database and Python-based fuzzy string matching libraries (FuzzyWuzzy), ElasticSearch provided the combination of features needed for our specific metadata harmonization tasks, particularly through its customizable field weighting and BM25 algorithm implementation.

For CDE generation, available resources were a significant consideration in deciding our methodology. The computational infrastructure required to host and operate state-of-the-art open-source LLMs, which requires specialized Graphical Processing Units (GPUs) hardware and technical expertise, was beyond our project’s scope and budget. A typical deployment of comparable models would require dedicated high-performance computing resources, costing thousands of dollars in hardware or cloud computing fees. The GPT-4 API offered a pragmatic alternative with manageable costs: our total API expenditure remained under $1000, allowing us to leverage advanced language model capabilities without significant infrastructure investment. This decision prioritized project feasibility and timely delivery over the potential benefits of operating our own models.

### Interoperability scoring

An Interoperability Scoring system was implemented to evaluate the effectiveness of CDE utilization and compatibility across datasets based on three criteria: Matching, Completeness, and Compliance. This provides analysts an easily interpretable metric to make decisions regarding data inclusion and could be used as a filtering criterion. It also permits the prioritization of resources and estimation of manual labor needed for data harmonization efforts. Ultimately, this scoring system is designed to assess a dataset’s readiness for integration into distributed research networks, including federated analyses where data are analyzed across institutions without being centrally pooled, as illustrated in Fig. 4^21,30^ .

Matching was evaluated based on the ability to map column headers from external sources to CDEs within our centralized repository using ElasticSearch. The descriptive and contextual metadata fields of generated CDEs were used for this task. The fields we included were the following: variable name, title, short description, preferred question text, and aliases. The percentage of external column headers correctly matched to the CDEs in the repository was calculated. If a data point could not be matched to an existing CDE, an expert reader evaluated whether a relevant CDE exists but was not matched or if an entirely new CDE should be generated.

Completeness was evaluated by determining the percentage of non-missing data fields for successfully mapped columns. Missing values can limit the ability to map data between columns, as these gaps mean some data points lack corresponding values for associative analysis.

Compliance was examined to ensure the values within non-null data points conformed to the permissible value sets associated with our generated CDEs. This criterion verified that data values within matched columns were recognized and fit within the acceptable ranges or categories as defined for each CDE. For continuous variables, the upper and lower ranges of values from the CDEs were used. For categorical variables (including ordinal variables redefined as categorical), compliance was based on the ability for a second round of BM25 ElasticSearch on parameters mirroring the initial matched phase.

These criteria collectively provided a comprehensive evaluation of dataset interoperability with the CDE repository, reflecting both the accuracy of data matching and the quality of data completeness and adherence to reporting conventions. The Interoperability Score is the percentage of values within a data field matched to a CDE that were not missing and within compliance, which is then averaged per data frame.

**Fig. 5:**
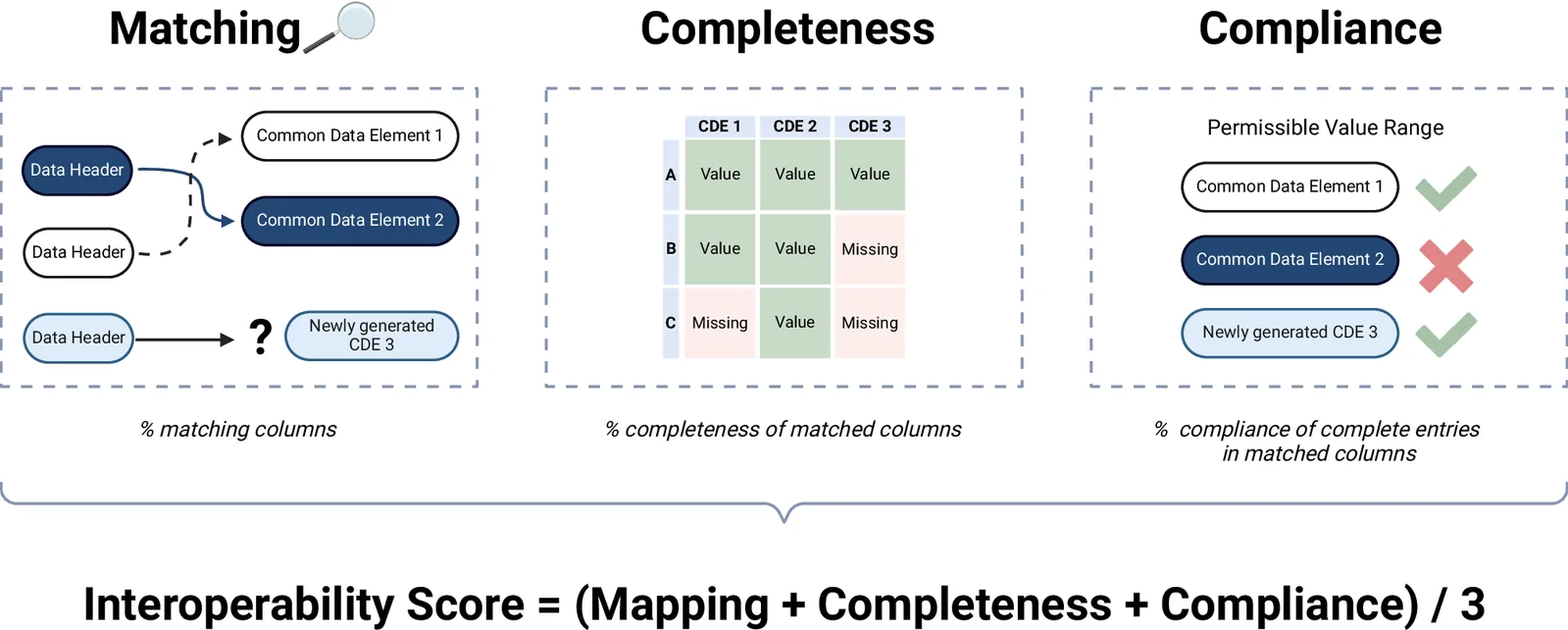
Interoperability scoring framework. This figure illustrates the three-component approach to quantifying cross-dataset compatibility. The interoperability score is calculated as the average of: 1) Matching - the percentage of column headers successfully mapped to existing CDEs; 2) Completeness - the percentage of non-null values in matched data fields; and 3) Compliance - the percentage of non-null values that conform to CDE-defined permissible ranges or categories. This assessment framework provides researchers with a standardized metric to evaluate dataset readiness for harmonization and integration into federated analyses. Created in BioRender. Jarreau, P. (2025) https://BioRender.com/g6gchj1.

The resulting Interoperability score formula is: *([% matching columns]* + *[% completeness of matched columns]* + *[% compliance of complete entries in matched columns])/3*. This will likely change as CDE databases expand and matching tools improve with time. Please see Fig. 5 for a summary of interoperability.

### Statistical validation of CDE generation consistency

To address the inherent probabilistic nature of large language models and validate the consistency of our CDE generation methodology, we conducted comprehensive semantic equivalence testing across multiple generation runs. We randomly selected 25 CDEs from our repository, representing diverse data types and sources, and regenerated them using identical prompts and parameters across multiple independent runs.

For each CDE, we evaluated semantic equivalence across four critical metadata components: variable name, title, description, and permissible values. Semantic equivalence was assessed using a binary scoring system (1 = semantically equivalent, 0 = not equivalent) through comprehensive contextual evaluation by domain experts. This evaluation considered whether regenerated components conveyed the same essential meaning and would function identically in a research context, even if exact wording differed.

We calculated component-level semantic equivalence rates as the mean equivalence score across all assessed CDEs, with standard errors computed to quantify variability. Cross-run consistency was determined by comparing equivalence patterns between Run 2 and Run 3 for each CDE component. Additionally, we categorized overall equivalence patterns into three groups: complete equivalence (all four components scored 1), partial equivalence (mixed scores), and no equivalence (all four components scored 0).

Statistical analyses were performed using Python 3.9 with numpy and pandas libraries. Visualization of results utilized matplotlib and seaborn for generation of publication-quality Figures, demonstrating both component-level performance and cross-run reproducibility.

## Supplementary information

Supplementary Information

## Data Availability

The datasets analyzed in this study are available from the following sources: BTRIS Clinical Observations data is accessible through https://btris.nih.gov/. CARD data resources can be accessed at https://card.nih.gov/data-resources/access-data. Clinical Trials Database Form Legends are available at https://ctdb.nichd.nih.gov/. Current Procedural Terminology and Healthcare Common Procedure Coding System (CPT-HCPCS) codes can be accessed via https://www.cms.gov/medicare/regulations-guidance/physician-self-referral/list-cpt-hcpcs-codes. Gastrointestinal Symptom Rating Scale (GSRS) information is described at https://pubmed.ncbi.nlm.nih.gov/3123181/. Health Rhythms and Inferences data is available from https://www.healthrhythms.com/. ICD coding data was obtained from https://www.cms.gov/medicare/coding-billing/icd-10-codes. Kubio HRV data specifications are available at https://www.kubios.com/. For datasets without publicly available URLs (Metabolic Cart, MNPQ Protocol, Ninehole Assessment, Resting State EEG, and Safety Monitor Survey), data dictionaries are available from the corresponding author upon reasonable request. A subset of the FAIRkit app's functionality used to view the initial CDE set built in this manuscript and referenced in this study is accessible at https://cde-browser-809832168532.us-central1.run.app/. An AI-enhanced evolution of this concept and over 1.3M CDEs can be found on our Hugging Face [https://huggingface.co/datasets/DataTecnica/RoP_biomedical].

## Code availability

The custom code used for AI-assisted CDE generation, matching, and interoperability scoring is publicly available at https://github.com/datatecnica/GenCDE. The code was implemented in Python with key dependencies including pandas, numpy, jinja2 (for templating), OpenAI API client (for embeddings and generation), rapidfuzz (for fuzzy matching), and rank_bm25 (for text relevance scoring). The matching pipeline uses a hybrid approach combining BM25, keyword matching, and vector similarity with Reciprocal Rank Fusion. Specific parameters for the BM25 algorithm (k1 = 1.2, b = 0.75) and GPT-4 model are detailed in the Methods section. The evolution of this code can also be found on our github at https://github.com/datatecnica/RoP_biomedical.

## References

[1] Pillai, A. S. & Menon, B. *Machine Learning and Deep Learning in Neuroimaging Data Analysis* (CRC Press, 2024).

[2] Li, Z., Jiang, X., Wang, Y. & Kim, Y. Applied machine learning in Alzheimer’s disease research: omics, imaging, and clinical data. *Emerg. Top. Life Sci.* **5**, 765–777 (2021). 10.1042/ETLS20210249 PMC8786302 34881778

[3] Fagnan, K. et al. *Data and Models: A Framework for Advancing AI in Science*. https://www.osti.gov/biblio/1579323 (2019).

[4] Scheffler, M. et al. FAIR data enabling new horizons for materials research. *Nature* **604**, 635–642 (2022). 10.1038/s41586-022-04501-x 35478233

[5] Adams, M. C. B., Hurley, R. W., Siddons, A., Topaloglu, U. & Wandner, L. D. NIH HEAL Clinical Data Elements (CDE) implementation: NIH HEAL Initiative IMPOWR network IDEA-CC. *Pain. Med.* **24**, 743–749 (2023). 10.1093/pm/pnad018 PMC10321760 36799548

[6] Kush, R. D. et al. FAIR data sharing: the roles of common data elements and harmonization. *J. Biomed. Inf.* **107**, 103421 (2020). 10.1016/j.jbi.2020.103421 32407878

[7] Rolland, B. et al. Toward rigorous data harmonization in cancer epidemiology research: one approach. *Am. J. Epidemiol.* **182**, 1033–1038 (2015). 10.1093/aje/kwv133 PMC4675662 26589709

[8] Balise, R. R. et al. Data cleaning and harmonization of clinical trial data: medication-assisted treatment for opioid use disorder. *PLoS One* **19**, e0312695 (2024). 10.1371/journal.pone.0312695 PMC11581333 39570967

[9] Torab-Miandoab, A., Samad-Soltani, T., Jodati, A. & Rezaei-Hachesu, P. Interoperability of heterogeneous health information systems: a systematic literature review. *BMC Med. Inf. Decis. Mak.* **23**, 18 (2023). 10.1186/s12911-023-02115-5 PMC9875417 36694161

[10] Varghese, J., Fujarski, M., Hegselmann, S., Neuhaus, P. & Dugas, M. CDEGenerator: an online platform to learn from existing data models to build model registries. *Clin. Epidemiol.* **10**, 961–970 (2018). 10.2147/CLEP.S170075 PMC6089100 30127646

[11] Acharya, D. & Mukhopadhyay, A. A comprehensive review of machine learning techniques for multi-omics data integration: challenges and applications in precision oncology. *Brief. Funct. Genomics* **23**, 549–560 (2024). 10.1093/bfgp/elae013 38600757

[12] Jiang, G. et al. Harmonization of detailed clinical models with clinical study data standards. *Methods Inf. Med.* **54**, 65–74 (2015). 10.3414/ME13-02-0019 25426730

[13] Sheehan, J. et al. Improving the value of clinical research through the use of Common Data Elements. *Clin. Trials* **13**, 671–676 (2016). 10.1177/1740774516653238 PMC5133155 27311638

[14] Petersen, R. C. et al. Alzheimer’s Disease Neuroimaging Initiative (ADNI): clinical characterization. *Neurology* **74**, 201 (2010). 10.1212/WNL.0b013e3181cb3e25 PMC2809036 20042704

[15] Global Parkinson’s Genetics Program GP2: The Global Parkinson’s Genetics Program. *Mov. Disord.* **36**, 842–851 (2021). 10.1002/mds.28494 PMC9290711 33513272

[16] Goldberger, A. L. et al. PhysioBank, PhysioToolkit, and PhysioNet: components of a new research resource for complex physiologic signals. *Circulation* **101**, E215–E220 (2000). 10.1161/01.cir.101.23.e215 10851218

[17] World Health Organization. *Th**e International Statistical Classification of Diseases and Health Related Problems ICD-10: Tenth Revision. Volume 2: Instruction Manual* (World Health Organization, 2004).

[18] Shahpori, R. & Doig, C. Systematized Nomenclature of Medicine-Clinical Terms direction and its implications on critical care. *J. Crit. Care* **25**, 364.e1–9 (2010). 10.1016/j.jcrc.2009.08.008 19836194

[19] Giannangelo, K. & Millar, J. Mapping SNOMED CT to ICD-10. In *Quality of Life through Quality of Information* 83–87 (IOS Press, 2012). 22874157

[20] Muludi, K., Fitria, K. M., Triloka, J. & Sutedi. Retrieval-augmented generation approach: document question answering using large language model. *Int. J. Adv. Comput. Sci*. **15**, 776–785 (2024).

[21] Han, H. et al. RAG vs. GraphRAG: A Systematic Evaluation and Key Insights. (2025).

[22] Kang, B., Yoon, J., Kim, H. Y., Jo, S. J., Lee, Y. & Kam, H. J. Deep-learning-based automated terminology mapping in OMOP-CDM. *J. Am. Med. Inform. Assoc.* **28**(7), 1489–1496 (2021). 10.1093/jamia/ocab030 PMC8279781 33987667

[23] Windisch, P. et al. The impact of temperature on extracting information from clinical trial publications using large language models. *Cureus* **16**, e75748 (2024). 10.7759/cureus.75748 PMC11731902 39811231

[24] Jinja Documentation. Jinja. https://jinja.palletsprojects.com/en/3.1.x/ (2022).

[25] OpenAI API Documentation -Structured Outputs. https://openai.com/index/introducing-structured-outputs-in-the-api/ (2024).

[26] ElasticSearch - Reading and writing documents. https://www.elastic.co/guide/en/elasticsearch/reference/current/docs-replication.html (2025).

[27] ElasticSearch - Relevance Tuning Guide, Weights and Boosts. https://www.elastic.co/guide/en/app-search/current/relevance-tuning-guide.html (2025).

[28] Camacho-Collados, J. & Pilehvar, M. T. Embeddings in natural language processing. In *Proceedings of the 28th International Conference on Computational Linguistics: Tutorial Abstracts* 10–15 (International Committee for Computational Linguistics, 2020).

[29] Reciprocal rank fusion outperforms condorcet and individual rank learning methods. 10.1145/1571941.1572114 (International Committee for Computational Linguistics, 2009).

[30] Jarreau, P. CDE Workflow. Created in BioRender. https://BioRender.com/r61p979 (2025).

[31] Jarreau, P. Interoperability Score. Created in BioRender. https://BioRender.com/g6gchj1 (2025).

