# Supplementary material for: A new AI assisted approach aligns data standards and accelerates interoperability in biomedical research
Source: NPJ Digit Med. 2026 Jun 12;9:733. doi: 10.1038/s41746-026-02795-z (PMC13616909; doi:10.1038/s41746-026-02795-z)
Supplement: Supplementary file 1 [file 41746_2026_2795_MOESM1_ESM.pdf]

Open Science Resources

Common data elements (CDEs) are housed for public access as part of a large data harmonization initiative at NIH. They are accessible through the FAIRkit (Findable, Accessible, Interoperable, Reusable toolkit) web app that can be found at https://cde-browser-809832168532.us-central1.run.app/. The source code is available at https://github.com/datatecnica/GenCDE.

The CDEs are accessible to the public without login. Just click the "General Users" identity tab, navigate to "query data" on the CDE tab. There are a total of over 43,000 human-in-the-loop validated CDEs available for data harmonization efforts on the FAIRkit web app (potentially the largest public repository of this sort), some of these relate to clinical data, EMR and REDCap related ontologies. To limit curated collections for sets of related CDEs, please filter by "collections" then select collections of interest in the drop down.

Specification and Cost Used for Running Models

We made use of the top performing LLM to date, OpenAI's GPT-4. Selection of model hyperparameters was to optimize performance. The temperature was set between 0.5 and 0.7. The maximum token limit was 100 tokens. According to OpenAI, a token is roughly equal to 4 characters and 100 tokens roughly equate to 75 words. We found this was a generous allotment for GPT responses with 13 tokens per response on average. The token limit still served to prevent potentially excessive responses. On average, 241 input tokens were used but approached 600 tokens in some cases. Token cost has come down significantly since we began and batch API request capability was released in April 2024, where token cost is reduced by half. This occurred near the end of CDE generation. To prime the GPT model to respond to these requests the following system prompt was used, "You are assisting in the standardization of clinical data dictionaries. Respond directly and concisely to each prompt, adhering to specified formats and contexts. Do not include labels, delimiters, or re-use the prompt in any way. Only the requested data". The system prompt was followed by 1 of 9 instructional prompts corresponding to different fields. An approximate cost breakdown of using GPT-4o to follow our methods detailed above is provided below.

Supplementary Table 1: Rough Estimate of Processing Costs During Study

Model	Input Token Cost	Output Token Cost	Avg. Input Tokens	Avg. Output Tokens	Approx. Cost Per Request + Response
GPT-4	\$0.0050 / 1k tokens	\$0.0150 / 1k tokens	241 tokens	13 tokens	\$0.0014

Prompt Templates for Common Data Element Generation

Overview

This document provides a comprehensive collection of prompt templates used in the AI-assisted Common Data Element (CDE) generation process. These templates were designed to systematically extract and standardize metadata across diverse biomedical datasets.

Template Design Principles

- Each template follows a consistent structure to ensure systematic metadata extraction
- Templates are tailored to specific metadata fields

- Contextual information can be conditionally included to enhance interpretation
- Output formats are explicitly defined to maintain consistency

Metadata Field Templates

1. Variable Name to Title Template

Purpose: Convert abbreviated or technical variable names into clear, descriptive titles

Jinja Template Content:

```
Instructions: Convert the following variable name into a clear,
descriptive title by expanding abbreviations.
### Data Element Details ###
Term:
"""
{{input_column}}
"""
{% if usr_added_prompt_txt %}
Additional context provided by the user:
{{ usr_added_prompt_txt }}
{% endif %}
{% if usr_selected_context_cols %}
Additional column data:
{% for col, value in usr_selected_context_cols.items() %}
- {{ col }}: {{ value }}
{% endfor %}
{% endif %}
```

2. Short Description Template

Purpose: Generate concise clinical explanations for data elements

Jinja Template Content:

```
Instructions: Respond with a precise and short clinical description
of the delimiter marked biomedical term below to enhance comprehension.
### Data Element Details ###
Term:
"""
{{input_column}}
"""
{% if usr_added_prompt_txt %}
Additional context provided by the user:
{{ usr_added_prompt_txt }}
{% endif %}
{% if usr_selected_context_cols %}
Additional column data:
{% for col, value in usr_selected_context_cols.items() %}
- {{ col }}: {{ value }}
{% endfor %}
{% endif %}
```

3. Preferred Question Text Template

Purpose: Create standardized assessment questions for data collection

Jinja Template Content:

```
Context: Assume the delimiter marked biomedical term is relevant to
a patient medical assessment.
### Data Element Details ###
Term:
"""
{{input_column}}
"""
Instructions: Form a question designed to acquire desired data or
information. If survey or questionnaire, use survey question text.
{% if usr_added_prompt_txt %}
Additional context provided by the user:
{{ usr_added_prompt_txt }}
{% endif %}
{% if usr_selected_context_cols %}
Additional column data:
{% for col, value in usr_selected_context_cols.items() %}
- {{ col }}: {{ value }}
{% endfor %}
{% endif %}
```

4. Permissible Values Template

Purpose: Establish standardized value sets for data elements

Jinja Template Content:

```
Instruction: Provide the permissible values for the following
biomedical term as a data element. Select the most appropriate
format based on the data type and context provided.
Available formats and when to use them:
1. **Numerical Interval** - For continuous numerical data:
   - Example: (Value1 - Value2)
2. **Categorical List** - For limited, specific options:
   - Example: Value1, Value2, Value3
3. **Binary Values** - For yes/no or true/false data only:
   - Example: True, False OR Yes, No
4. **Dictionary** - For key-value pairs:
   - Format as "Key1: Value1, Key2: Value2"
5. **Free Text** - ONLY as a last resort:
   - Example: free text
### Data Element Details ###
Term:
"""
{{input_column}}
"""
{% if value_format %}
Value Format: {{ value_format }}
{% endif %}
{% if unit_of_measure %}
Unit of Measure: {{ unit_of_measure }}
{% endif %}
{% if minimum_value %}
Minimum Value: {{ minimum_value }}
{% endif %}
{% if maximum_value %}
Maximum Value: {{ maximum_value }}
```

```
{% endif %}
{% if usr_added_prompt_txt %}
Additional context provided by the user:
{{ usr_added_prompt_txt }}
{% endif %}
{% if usr_selected_context_cols %}
Additional column data:
{% for col, value in usr_selected_context_cols.items() %}
{{ col }}: {{ value }}
{% endfor %}
{% endif %}
```

5. Unit of Measure Template

Purpose: Define a standard unit of measure for a data element

Jinja Template Content:

```
Instruction: Based on the data element title below, specify the
appropriate unit of measurement. Consider any context provided.
Format your response as a concise unit specification.
### Data Element Details ###
Term:
"""
{{input_column}}
"""
{% if value_format %}
Value Format: {{ value_format }}
{% endif %}
{% if permissible_values %}
Permissible Values: {{ permissible_values }}
{% endif %}
{% if preferred_question_text %}
Preferred Question Text: {{ preferred_question_text }}
{% endif %}
{% if usr_added_prompt_txt %}
Additional context provided by the user:
{{ usr_added_prompt_txt }}
{% endif %}
{% if usr_selected_context_cols %}
Additional column data:
{% for col, value in usr_selected_context_cols.items() %}
- {{ col }}: {{ value }}
{% endfor %}
{% endif %}
```

6. Aliases Template

Purpose: Collect potential text strings associated with data element to enhance semantic depth of the CDE for mapping purposes.

Jinja Template Content:

```
Instructions: Based on the existing title for the data element
below and the provided context, create a comma-separated list of
appropriate alternative aliases and names found in medical
ontologies, terminologies, vocabularies, taxonomies, and coding
systems. Prioritize accurate, applicable, and specific
related-terms over generic. Emphasis on directly applicable.
### Data Element Details ###
Term:
"""
{{input_column}}
"""
### Context ###
{% if usr_added_prompt_txt %}
Additional context provided by the user:
{{ usr_added_prompt_txt }}
{% endif %}
{% if usr_selected_context_cols %}
Additional column data:
{% for col, value in usr_selected_context_cols.items() %}
- {{ col }}: {{ value }}
{% endfor %}
{% endif %}
```

7. Variable Name / Header Template

Purpose: Creating abbreviated column headers for CDEs that were generated from concept codes (e.g. ICD code – G20.A – Parkinson's disease without dyskinesia)

Jinja Template Content:

```
Instructions: Generate a standardized variable name for the data
element below. Use snake_case, be concise, and ensure the name
is suitable for data systems. Do not include any labels or
additional text in your response.
### Data Element Details ###
Term:
"""
{{input_column}}
"""
{% if usr_added_prompt_txt %}
Additional context provided by the user:
{{ usr_added_prompt_txt }}
{% endif %}
{% if usr_selected_context_cols %}
Additional column data:
{% for col, value in usr_selected_context_cols.items() %}
- {{ col }}: {{ value }}
{% endfor %}
{% endif %}
```

Methodology Notes

- Templates were applied iteratively, with each template building upon the output of previous templates
- Subject matter experts reviewed and refined generated metadata
- Contextual information was crucial for accurate interpretation of abbreviated or technical terms
- This generic system message was attached to each OpenAI API request defining the role and context: "You are assisting in the standardization of clinical data dictionaries. Respond directly and concisely to each prompt, adhering to specified formats and contexts. Do not include labels, delimiters, or re-use prompt in any way."

Limitations and Considerations

- Template effectiveness varied based on source data complexity
- Zero-shot prompting was primarily used to manage token costs and avoid bias from example-based learning

Supplementary Table 2: Reproducibility Test – Run 1

original variable name	original title	original short description	original permissible values	new title	new short description	new permissible values	new variable name	title equivalent	description equivalent	values equivalent	varname equivalent
nlm_gds_long_form_clear_mind_ind	Geriatric Depression Scale (GDS) Long Form -Clear mind indicator	The Geriatric Depression Scale (GDS) Long Form - Clear mind indicator is a measu...	Yes:Yes.No:No. Unknown: Unknown	National Library of Medicine General Distress Scale Long Form Clear Mind Indicat...	The National Library of Medicine General Distress Scale Long Form Clear Mind Ind...	List:Never, Rarely, Sometimes, Often,Very Often	nml_gd_scale_lf_clear_mind_indicator	0	0	0	1
aire mutation	AIRE MUTATION	AIRE mutation refers to genetic alterations in the autoimmune regulator (AIRE) g...	True ,False	Autoimmune Regulator Mutation	A genetic change in the Autoimmune Regulator (AIRE) gene which plays a role in i...	Homozygous, Heterozygous, Compound Heterozygous,No Mutation Detected	autoimmune_regulator_mutation	1	1	0	1
other organ or tissue replaced by other means	Other Organ or Tissue Replaced by Other Means	This term refers to the surgical or medical replacement of an organ or tissue us...	True ,False	Other Organ or Tissue Replaced by Other Methods	This term refers to the clinical procedure of replacing an organ or tissue in th...	Yes,No	other_organ_tissue_replaced_other_methods	1	1	1	1
nlm_molar_ratio_alo_iso_leu_hyp_ala_dbs	Molar Ratio of Alloisoleucine, Isoleucine,Leucine, Hydroxyproline to Alanine in...	Alloisoleucine+ Isoleucine+Leucine+ Hydroxyproline/ Alanine is a primary marker for...	min:0,max:100, unit:ratio	National Library of Medicine Molar Ratio of Allopurinol, Isoleucine,Leucine, Hy...	This term refers to the proportion of Allopurinol, Isoleucine,Leucine, Hydroxyp...	Range:min: 0,max:1, unit:Moles/ Litre	nml_molar_ratio_allopurinol_isoleucine_leucine_hydroxyproline_alanine_deriv_bloo...	0	0	0	0
squamous_blepharitis_rt_l	Squamous blepharitis right lower eyelid	Inflammatory condition with squamous scaling affecting the right lower eyelid,o...	0:No—1:Yes	Squamous Blepharitis Right Lower Tract	Inflammation of the squamous cells in the right lower eyelid area.	Present, Absent	sq_blepharitis_r_lower_tract	0	0	0	0
nlm_med_exp_date	Medication Expiration Date	The Medication Expiration Date refers to the specific day after which a medicati...	yyyy-mm-dd	National Library of Medicine Medication Expiration Date	The National Library of Medicine Medication Expiration Date is the specific date...	Format: Date Time (YYYY-MM-DD) Example: 2023-06-20	nml_med_exp_date	1	0	0	0
bilateral_eye_injuries	Localized traumatic opacities,bilateral	Indicates the presence of localized opacities in both eyes resulting from trauma...	[{"value":0, "labels":["No", "False", "Absent"]}, {"value":1, "labels":["Yes...	Bilateral Eye Injuries	Injuries that have occurred on both eyes.	Yes,No	bilateral_eye_injuries	0	0	1	1

Supplementary Table 2 (continued)

original variable name	original title	original short description	original permissible values	new title	new short description	new permissible values	new variable name	title equivalent	description equivalent	values equivalent	varname equivalent
ast_bld	Aspartate Transaminase Blood Level	The Aspartate Transaminase Blood Level refers to the concentration of the AST en...	(y¿0)&(y¡ 10000)	Asthma Blood Test	A diagnostic procedure that analyzes a patient's blood sample for various marker...	Unrestricted Text	asthma_blood_test	0	0	0	1
dyskeratosis congenita autosomal recessive	Dyskeratosis congenita autosomal recessive	Dyskeratosis congenita autosomal recessive is a severe,inherited disorder chara...	True ,False	Dyskeratosis Congenita Autosomal Recessive Type	Dyskeratosis Congenita Autosomal Recessive Type refers to a rare form of Dyskera...	DKC1 mutation, TERC mutation, TERT mutation, NOP10 mutation, NHP2 mutation, WRAP...	dyskeratosis_congenita_autosomal_recessive_type	1	1	0	0
truncal_lymphadenitis	Acute lymphadenitis of trunk	Rapid onset inflammation of lymph nodes in the trunk region accompanied by pain,...	0:No—1:Yes	Truncal Lymphadenitis	Truncal lymphadenitis refers to an inflammatory condition involving lymph nodes ...	Yes,No	truncal_lymphadenitis	1	0	1	1
paternal_grandfather_birth_place	Paternal Grandfather's Place of Birth	The location where the patient's paternal grandfather was born.	free text	Paternal Grandfather's Place of Birth	The location where the father's father was born.	Unrestricted Text	paternal_grandfather_birth_place	1	1	1	1
rt_eye_chorioretinal_lesi	Unspecified focal chorioretinal inflammation,right eye	Localized inflammatory lesion in the chorioretinal tissue of the right eye with ...	0:No—1:Yes	Right Eye Chorioretinal Lesion	A disease condition characterized by damage or abnormalities in the choroid and ...	Presence: Yes,No Size: min:0,max: 30,unit:mm Localization: Macular, Perip...	right_eye_chorioretinal_lesion	0	0	0	1
halluc_dep_psych_delus	Hallucinogen dependence w psychotic disorder w delusions	Indicates the presence of dependence on hallucinogens accompanied by psychotic f...	0:No—1:Yes	Hallucination Dependence in Psychiatric Delusions	Hallucination Dependence in Psychiatric Delusions refers to a condition where a ...	Not at all dependent, Mildly dependent, Moderately dependent, Highly dependent	hallucination_dependence_psych_delusions	0	0	0	1

Supplementary Table 2 (continued)

original variable name	original title	original short description	original permissible values	new title	new short description	new permissible values	new variable name	title equivalent	description equivalent	values equivalent	varname equivalent
nlm_d_dimer_assay_used	D-Dimer Commercial Assay Used,D-dimer Assay	The D-Dimer Commercial Assay refers to the specific commercial product used to c...	Technoclone TechnoLEIA D-Dimer Latex Kit:Technoclone TechnoLEIA D-Dimer Latex K...	National Library of Medicine D-Dimer Assay Used	The National Library of Medicine D-Dimer Assay Used is a test implemented to det...	Manufacturer1: STA Liatest D-DI, Manufacturer2: Innovance D-Dimer, Manufacturer3...	nat_lib_med_d_dimer_assay_used	0	0	0	1
public_speaking	Public speaking	Anxiety related to public speaking, characterized by intense fear or avoidance o...	0:No,1:Yes	Public Speaking	Public speaking refers to the process of communicating information to a group of...	Unrestricted Text	public_speaking	1	0	0	1
mottled_teeth	Mottled teeth	Displays dental enamel with irregular discoloration,which may indicate developm...	0:No—1:Yes	Presence of Mottled Teeth	Mottled Teeth refers to a condition characterized by irregular,patchy discolora...	Yes,No	mottled_teeth_presence	1	0	1	0
seps_strep	Sepsis due to streptococcus, group B	Indicates the presence or absence of sepsis caused by Group B Streptococcus,con...	True/False OR 0/1	Sepsis due to Streptococcus	Sepsis caused by a bacterial infection from the Streptococcus species.	Yes,No	sepsis_due_to_streptococcus	0	0	1	1
insomnia_due_other_mental	Insomnia due to other mental disorder	Indicates whether the patient experiences insomnia attributable to a concomitant...	0:No—1:Yes	Insomnia Due to Other Mental Conditions	Insomnia Due to Other Mental Conditions is a type of sleep disorder where an ind...	Yes,No	insomnia_due_mental_conditions	1	1	1	1
serpiginous chorioretinopathy	SERPIGINOUS CHORIORETINOPATHY	Seripiginous Chorioretinopathy is a rare,chronic inflammatory disease causing p...	True ,False	Serpiginous Choroidoretinopathy	Serpiginous Choroidoretinopathy is a rare,chronic inflammatory disease that aff...	Present,Not Present	serpiginous_choroidoretinopathy	1	1	0	0
ankle swelling	ANKLE SWELLING	Ankle swelling refers to an abnormal enlargement or inflation of the ankle due t...	True ,False	Presence of Ankle Swelling	Ankle swelling refers to the condition where there's an increase in size or puff...	Yes,No	ankle_swelling_presence	1	1	1	1
nlm_part_blast_sensor_acc_samp_rate	Participant's Blast Sensor Acceleration Sampling Rate	The participant/subject's blast sensor acceleration sampling rate.	min:0,max:10000,unit:Hz	National Library of Medicine Particle Blast Sensor Accelerometer Sample Rate	The National Library of Medicine Particle Blast Sensor Accelerometer Sample Rate...	min:1 Hz, max:5 kHz, unit:Hertz	nml_particle_blast_sensor_accel_samp_rate	0	0	0	0

Supplementary Table 2 (continued)

original variable name	original title	original short description	original permissible values	new title	new short description	new permissible values	new variable name	title equivalent	description equivalent	values equivalent	varname equivalent
cgd relative	CGD RELATIVE	CGD Relative refers to a family member of an individual diagnosed with Chronic G...	True ,False	Congenital Generalized Disease Relative	A familial or hereditary disease present from birth that affects multiple system...	Yes,No	cong_gen_dis_rel	0	0	1	1
local excision or destruction of lesion or tissue of trachea	Local Excision or Destruction of Lesion or Tissue of Trachea	This refers to a surgical procedure that involves removing or destroying abnorm...	True ,False	Local Excision or Destruction of Lesion or Tissue of Trachea	This is a medical procedure where a specific abnormality (lesion) or tissue in t...	Yes,No	local_excision_destruction_lesion_trachea	1	1	1	1
nlm_hemiplegia_lat_type	Hemiplegia laterality type	Hemiplegia laterality type refers to the side of the body (left or right) that i...	Left:Left.Right: Right	National Library of Medicine Hemiplegia Laterality Type	The National Library of Medicine Hemiplegia Laterality Type refers to a classifi...	Left,Right, Bilateral	nml_hemiplegia_laterality_type	1	1	0	0
chronic_gout_due_renal_im	Chronic gout due to renal impairment,right hip,with tophus	Chronic gout related to renal dysfunction affecting the right hip, characterized...	0:No—1:Yes	Chronic Gout Due to Renal Impairment	Chronic Gout Due to Renal Impairment refers to a long-standing condition charact...	Yes,No	chronic_gout_due_renal_impairment	0	0	1	1

Supplementary Table 3: Reproducibility Test – Run 2

**original variable name**	**original title**	**original short description**	**original permissible values**	**new title**	**new short description**	**new permissible values**	**new variable name**	**title equivalent**	**description equivalent**	**values equivalent**	**varname equivalent**
nlm_gds_long_form_clear_mind_ind	Geriatric Depression Scale (GDS) Long Form -Clear mind indicator	The Geriatric Depression Scale (GDS) Long Form - Clear mind indicator is a measu...	Yes:Yes.No:No. Unknown: Unknown	National Library of Medicine General Deterioration Scale Long Form Clear Mind In...	The National Library of Medicine General Deterioration Scale Long Form Clear Min...	Yes,No	nml_gen_det_scale_lf_clear_mind_ind	0	0	1	0
aire mutation	AIRE MUTATION	AIRE mutation refers to genetic alterations in the autoimmune regulator (AIRE) g...	True ,False	Autoimmune Regulator Gene Mutation	A change or alteration in the Autoimmune Regulator (AIRE) gene that disrupts its...	Present:Yes, Absent:No	auto_reg_gene_mut	1	1	1	1
other organ or tissue replaced by other means	Other Organ or Tissue Replaced by Other Means	This term refers to the surgical or medical replacement of an organ or tissue us...	True ,False	Other Organ or Tissue Replaced by Other Means	This term refers to a medical procedure where an organ or tissue is substituted ...	Heart, Lungs, Kidneys, Liver, Pancreas, Intestines, Stomach, Esophagus, Bladder,...	organ_tissue_replaced_other_means	1	1	0	1
nlm_molar_ratio_alo_iso_leu_hyp_ala_dbs	Molar Ratio of Alloisoleucine, Isoleucine,Leucine, Hydroxyproline to Alanine in...	Alloisoleucine+ Isoleucine+Leucine+ Hydroxyproline/ Alanine is a primary marker for...	min:0,max:100, unit:ratio	National Library of Medicine Molar Ratio of Alanine, Isoleucine,Leucine, Hydrox...	The National Library of Medicine Molar Ratio of Alanine,Isoleucine,Leucine, Hy...	Range:min: 0,max:100, unit: molar%	nml_molar_ratio_alanine_isoleucine_leucine_hydroxyproline_alanyl_dibasic_salt	0	0	1	0
squamous_blepharitis_rt_l	Squamous blepharitis right lower eyelid	Inflammatory condition with squamous scaling affecting the right lower eyelid,o...	0:No—1:Yes	Squamous Blepharitis Right Lower Lid	Inflammation of the right lower eyelid,characterized by the presence of squamou...	Not Present, Mild, Moderate, Severe	sqamous_blepharitis_rgt_lwr_lid	1	1	0	1
nlm_med_exp_date	Medication Expiration Date	The Medication Expiration Date refers to the specific day after which a medicati...	yyyy-mm-dd	National Library of Medicine Medication Expiration Date	The date specified by the National Library of Medicine indicating when a specifi...	Range:min: 01/01/1900, max: Current Date,unit: Date (mm/ dd/yyyy)	nml_med_expiration_date	1	0	1	0
bilateral_eye_injuries	Localized traumatic opacities,bilateral	Indicates the presence of localized opacities in both eyes resulting from trauma...	[{"value":0, "labels":["No", "False", "Absent"]}, {"value":1, "labels":["Yes...	Bilateral Eye Injuries	Injuries that have occurred to both eyes.	Yes,No	bilat_eye_injuries	0	0	1	1

Supplementary Table 3 (continued)

original variable name	original title	original short description	original permissible values	new title	new short description	new permissible values	new variable name	title equivalent	description equivalent	values equivalent	varname equivalent
ast_bld	Aspartate Transaminase Blood Level	The Aspartate Transaminase Blood Level refers to the concentration of the AST en...	(y¿0)&(y¡ 10000)	Asthma Blood Test	A laboratory test performed to evaluate the levels of certain antibodies produce...	List: Negative, Borderline, Positive	asthma_blood_test	0	0	0	0
dyskeratosis congenita autosomal recessive	Dyskeratosis congenita autosomal recessive	Dyskeratosis congenita autosomal recessive is a severe,inherited disorder chara...	True ,False	Dyskeratosis Congenita with Autosomal Recessive Inheritance	Dyskeratosis Congenita with Autosomal Recessive Inheritance is a rare genetic di...	Confirmed, Carrier, Non-carrier	dyskeratosis_congenita_autosomal_recessive_inheritance	1	1	0	1
truncal_lymphadenitis	Acute lymphadenitis of trunk	Rapid onset inflammation of lymph nodes in the trunk region accompanied by pain,...	0:No—1:Yes	Truncal Lymphadenitis	Truncal lymphadenitis is a medical condition characterized by the inflammation o...	Present, Absent	truncal_lymphadenitis	1	1	1	1
paternal_grandfather_birth_place	Paternal Grandfather's Place of Birth	The location where the patient's paternal grandfather was born.	free text	Paternal Grandfather's Place of Birth	The location where the father's father of an individual was born.	Unrestricted Text	paternal_grandfather_birth_place	1	1	1	1
rt_eye_chorioretinal_lesi	Unspecified focal chorioretinal inflammation,right eye	Localized inflammatory lesion in the chorioretinal tissue of the right eye with ...	0:No—1:Yes	Right Eye Chorioretinal Lesion	A chorioretinal lesion is a type of eye condition affecting the choroid layer or...	Present,Not Present	right_eye_chorioretinal_lesion	1	1	1	1
halluc_dep_psych_delus	Hallucinogen dependence w psychotic disorder w delusions	Indicates the presence of dependence on hallucinogens accompanied by psychotic f...	0:No—1:Yes	Hallucination Dependency Psychiatric Delusion	Hallucination Dependency Psychiatric Delusion refers to a mental health disorder...	True,False	halluc_dep_psych_delusion	0	0	1	1
nlm_d_dimer_assay_used	D-Dimer Commercial Assay Used,D-dimer Assay	The D-Dimer Commercial Assay refers to the specific commercial product used to c...	Technoclone TechnoLEIA D-Dimer Latex Kit:Technoclone TechnoLEIA D-Dimer Latex K. ..	National Library of Medicine D-Dimer Assay Used	A test utilized by the National Library of Medicine that measures the amount of ...	List: Immunoassay, Quantitative enzyme, Immunoturbidimetric assay, Microplate en...	natl_lib_med_ddimer_assay_used	1	0	0	1
public_speaking	Public speaking	Anxiety related to public speaking, characterized by intense fear or avoidance o...	0:No,1:Yes	Public Speaking	Public Speaking refers to the act of delivering a speech or presentation to a li...	Unrestricted Text	public_speaking	1	0	0	1

Supplementary Table 3 (continued)

original variable name	original title	original short description	original permissible values	new title	new short description	new permissible values	new variable name	title equivalent	description equivalent	values equivalent	varname equivalent
mottled_teeth	Mottled teeth	Displays dental enamel with irregular discoloration,which may indicate developm...	0:No—1:Yes	Presence of Mottled Teeth	Presence of Mottled Teeth refers to a dental condition characterized by irregula...	Yes,No	mottled_teeth_presence	1	1	1	1
seps_strep	Sepsis due to streptococcus, group B	Indicates the presence or absence of sepsis caused by Group B Streptococcus,con...	True/False OR 0/1	Sepsis due to Streptococcus	Sepsis is a systemic,often life-threatening response to an infection,in this c...	Suspected, Confirmed, Not Present	sepsis_due_to_streptococcus	0	0	1	1
insomnia_due_other_mental	Insomnia due to other mental disorder	Indicates whether the patient experiences insomnia attributable to a concomitant...	0:No—1:Yes	Insomnia Due to Other Mental Conditions	Insomnia Due to Other Mental Conditions refers to the sleep disorder characteriz...	Yes,No	insomnia_due_mental_conditions	1	1	1	1
serpiginous chorioretinopathy	SERPIGINOUS CHORIORETINOPATHY	Seripiginous Chorioretinopathy is a rare,chronic inflammatory disease causing p...	True ,False	Serpiginous Chorioretinitis Disease Status	Serpiginous Chorioretinitis Disease Status refers to the current condition of a ...	Active,Non-Active,In Remission	serpiginous_chorioretinitis_disease_status	1	1	0	0
ankle swelling	ANKLE SWELLING	Ankle swelling refers to an abnormal enlargement or inflation of the ankle due t...	True ,False	Ankle Swelling Presence	Indicates whether or not there is an increase in the size or a change in the sha...	Yes,No	ankle_swell_presence	1	1	1	1
nlm_part_blast_sensor_acc_samp_rate	Participant's Blast Sensor Acceleration Sampling Rate	The participant/subject's blast sensor acceleration sampling rate.	min:0,max:10000,unit:Hz	National Library of Medicine Particle Blast Sensor Accelerometer Sample Rate	The National Library of Medicine Particle Blast Sensor Accelerometer Sample Rate...	min:1 Hz, max:5000 Hz,unit: Hertz	nml_particle_blast_sensor_accel_sample_rate	1	0	1	0
cgd relative	CGD RELATIVE	CGD Relative refers to a family member of an individual diagnosed with Chronic G...	True ,False	Chronic Granulomatous Disease Relative	A family member who has Chronic Granulomatous Disease,a rare disorder that affe...	First Degree, Second Degree, Third Degree,Not Related	chronic_granulomatous_disease_relative	1	0	1	1
local excision or destruction of lesion or tissue of trachea	Local Excision or Destruction of Lesion or Tissue of Trachea	This refers to a surgical procedure that involves removing or destroying abnorm...	True ,False	Local Excision or Destruction of Lesion or Tissue of Trachea	A medical procedure where a lesion or tissue in the trachea is surgically remove...	Yes,No	local_excision_destruction_lesion_tissue_trachea	1	1	1	1
nlm_hemiplegia_lat_type	Hemiplegia laterality type	Hemiplegia laterality type refers to the side of the body (left or right) that i...	Left:Left.Right:Right	National Library of Medicine Hemiplegia Laterality Type	The National Library of Medicine Hemiplegia Laterality Type is a medical term in...	List:Left, Right, Bilateral	nml_hemiplegia_laterality_type	1	1	0	1

Supplementary Table 3 (continued)

original variable name	original title	original short description	original permissible values	new title	new short description	new permissible values	new variable name	title equivalent	description equivalent	values equivalent	varname equivalent
chronic_gout_due_renal_im	Chronic gout due to renal impairment,right hip,with tophus	Chronic gout related to renal dysfunction affecting the right hip, characterized...	0:No—1:Yes	Chronic Gout Due to Renal Impairment	Chronic Gout Due to Renal Impairment is a persistent condition where high levels...	True,False	chronic_gout_due_renal_impairment	1	0	1	1

Supplementary Table 4: Reproducibility Test – Run 3

original variable name	original title	original short description	original permissible values	new title	new short description	new permissible values	new variable name	title equivalent	description equivalent	values equivalent	varname equivalent
nlm_gds_long_form_clear_mind_ind	Geriatric Depression Scale (GDS) Long Form -Clear mind indicator	The Geriatric Depression Scale (GDS) Long Form - Clear mind indicator is a measu...	Yes:Yes.No:No. Unknown: Unknown	National Library of Medicine Genetic Data Sharing Indicator for Clear Mind Long ...	The National Library of Medicine Genetic Data Sharing Indicator for Clear Mind L...	Yes,No	nml_genetic_data_sharing_ind_clear_mind_lf	0	0	1	0
aire mutation	AIRE MUTATION	AIRE mutation refers to genetic alterations in the autoimmune regulator (AIRE) g...	True ,False	Autoimmune Regulator Gene Mutation	A genetic alteration in the Autoimmune Regulator (AIRE) gene causing the body's ...	Positive, Negative	autoimmune_regulator_gene_mutation	1	1	1	1
other organ or tissue replaced by other means	Other Organ or Tissue Replaced by Other Means	This term refers to the surgical or medical replacement of an organ or tissue us...	True ,False	Other Organ or Tissue Replaced by Other Methods	This term refers to a procedure where a particular organ or tissue in the body i...	List:-Heart -Kidney - Liver - Pancreas - Lung - Intestine - Uterus - Vascula...	organ_tissue_replaced_other_methods	1	1	0	1
nlm_molar_ratio_alo_iso_leu_hyp_ala_dbs	Molar Ratio of Alloisoleucine, Isoleucine,Leucine, Hydroxyproline to Alanine in...	Alloisoleucine+ Isoleucine+Leucine+ Hydroxyproline/ Alanine is a primary marker for...	min:0,max:100, unit:ratio	National Library of Medicine Molar Ratio of Alanine, Isoleucine,Leucine, Hydrox...	This term refers to a specific ratio derived from the National Library of Medici...	min:0,max: 1,unit:molar ratio	nml_molar_ratio_aln_isln_leu_hydroxypro_albumin_dbl_bnd_struct	0	0	1	0
squamous_blepharitis_rt_l	Squamous blepharitis right lower eyelid	Inflammatory condition with squamous scaling affecting the right lower eyelid,o...	0:No—1:Yes	Squamous Blepharitis Right Lower	Inflammation of the outer skin layer of the right lower eyelid.	Yes,No	sq_blepharitis_r_lower	1	0	1	1
nlm_med_exp_date	Medication Expiration Date	The Medication Expiration Date refers to the specific day after which a medicati...	yyyy-mm-dd	National Library of Medicine Medication Expiration Date	The date when a medication,as cataloged by the National Library of Medicine,is...	min:01/01/ 1900 ,max: present date ,unit:mm/ dd/yyyy	nml_med_expiration_date	1	1	1	0
bilateral_eye_injuries	Localized traumatic opacities,bilateral	Indicates the presence of localized opacities in both eyes resulting from trauma...	[{"value":0, "labels":["No", "False", "Absent"]}, {"value":1, "labels":["Yes...	Bilateral Eye Injuries	Injuries that occur in both eyes simultaneously.	Yes,No	bilat_eye_injuries	0	0	1	1
ast_bld	Aspartate Transaminase Blood Level	The Aspartate Transaminase Blood Level refers to the concentration of the AST en...	(y¿0)&(y¡ 10000)	Aspartate Aminotransferase Blood Level	Aspartate Aminotransferase Blood Level is a diagnostic measure indicating the am. ..	min:0,max: 200,unit:U/ L	asp_aminotrans_blood_lvl	1	1	1	1

Supplementary Table 4 (continued)

original variable name	original title	original short description	original permissible values	new title	new short description	new permissible values	new variable name	title equivalent	description equivalent	values equivalent	varname equivalent
dyskeratosis congenita autosomal recessive	Dyskeratosis congenita autosomal recessive	Dyskeratosis congenita autosomal recessive is a severe,inherited disorder chara...	True ,False	Dyskeratosis Congenita with Autosomal Recessive Inheritance Pattern	Dyskeratosis Congenita with Autosomal Recessive Inheritance Pattern is a rare ge...	Positive, Negative, Unknown	dyskeratosis_congenita_autosomal_recessive_inheritance	1	1	0	1
truncal_lymphadenitis	Acute lymphadenitis of trunk	Rapid onset inflammation of lymph nodes in the trunk region accompanied by pain,...	0:No—1:Yes	Truncal Lymphadenitis	Truncal Lymphadenitis refers to the inflammation of lymph nodes located in the t...	Yes,No	truncal_lymphadenitis	1	1	1	1
paternal_grandfather_birth_place	Paternal Grandfather's Place of Birth	The location where the patient's paternal grandfather was born.	free text	Paternal Grandfather's Place of Birth	The location or city where the father's father of a patient was born.	Unrestricted Text	paternal_grandfather_birth_place	1	1	1	1
rt_eye_chorioretinal_lesi	Unspecified focal chorioretinal inflammation,right eye	Localized inflammatory lesion in the chorioretinal tissue of the right eye with ...	0:No—1:Yes	Right Eye Chorioretinal Lesion	A right eye chorioretinal lesion refers to an abnormal tissue or growth in the c...	Present, Absent	right_eye_chorioretinal_lesion	1	0	1	1
halluc_dep_psych_delus	Hallucinogen dependence w psychotic disorder w delusions	Indicates the presence of dependence on hallucinogens accompanied by psychotic f...	0:No—1:Yes	Hallucinatory Dependency in Psychiatric Delusions	Hallucinatory Dependency in Psychiatric Delusions refers to a condition in menta...	List:Very Low,Low, Moderate, High,Very High	hallucinatory_dependency_psychiatric_delusions	0	0	1	1
nlm_d_dimer_assay_used	D-Dimer Commercial Assay Used,D-dimer Assay	The D-Dimer Commercial Assay refers to the specific commercial product used to c...	Technoclone TechnoLEIA D-Dimer Latex Kit:Technoclone TechnoLEIA D-Dimer Latex K...	National Library of Medicine Delta Dimer Assay Used	The National Library of Medicine Delta Dimer Assay Used is a standardized testin...	Yes,No	nml_delta_dimer_assay_used	1	0	0	1
public_speaking	Public speaking	Anxiety related to public speaking, characterized by intense fear or avoidance o...	0:No,1:Yes	Public Speaking	The act of delivering a speech or presentation to a live audience in a structure...	List:Not at all comfortable, Somewhat comfortable, Neutral, Somewhat uncomforta...	public_speaking	1	0	1	1
mottled_teeth	Mottled teeth	Displays dental enamel with irregular discoloration,which may indicate developm...	0:No—1:Yes	Presence of Mottled Teeth	Mottled teeth refer to a dental condition characterized by irregular spots,stre...	Yes,No	mottled_teeth_presence	1	0	1	1

Supplementary Table 4 (continued)

original variable name	original title	original short description	original permissible values	new title	new short description	new permissible values	new variable name	title equivalent	description equivalent	values equivalent	varname equivalent
seps_strep	Sepsis due to streptococcus, group B	Indicates the presence or absence of sepsis caused by Group B Streptococcus,con...	True/False OR 0/1	Sepsis due to Streptococcus	Sepsis,a severe and potentially life-threatening condition triggered by an infe...	Yes,No	sepsis_due_to_streptococcus	0	0	1	1
insomnia_due_other_mental	Insomnia due to other mental disorder	Indicates whether the patient experiences insomnia attributable to a concomitant...	0:No—1:Yes	Insomnia Due to Other Mental Conditions	Insomnia Due to Other Mental Conditions refers to a sleep disorder characterized...	Yes,No	insomnia_due_mental_conditions	1	1	1	1
serpiginous chorioretinopathy	SERPIGINOUS CHORIORETINOPATHY	Seripiginous Chorioretinopathy is a rare,chronic inflammatory disease causing p...	True ,False	Serpiginous Chorioretinopathy Condition Status	Serpiginous Chorioretinopathy Condition Status refers to the current state or pr...	Yes,No	serpiginous_chorioretinopathy_cond_status	1	1	1	1
ankle swelling	ANKLE SWELLING	Ankle swelling refers to an abnormal enlargement or inflation of the ankle due t...	True ,False	Presence of Ankle Swelling	Presence of Ankle Swelling refers to a clinical condition where the ankle area e...	Yes,No	ankle_swelling_presence	1	1	1	1
nlm_part_blast_sensor_acc_samp_rate	Participant's Blast Sensor Acceleration Sampling Rate	The participant/subject's blast sensor acceleration sampling rate.	min:0,max:10000,unit:Hz	National Library of Medicine Particle Blast Sensor Acceleration Sample Rate	The National Library of Medicine Particle Blast Sensor Acceleration Sample Rate ...	min:100 Hz, max:10000 Hz,unit: Hertz	nml_particle_blast_sensor_accel_sample_rate	1	0	1	0
cgd relative	CGD RELATIVE	CGD Relative refers to a family member of an individual diagnosed with Chronic G...	True ,False	Congenital Granulomatous Disease Relative	Congenital Granulomatous Disease Relative refers to a family member who has a he...	Yes,No	congenital_granulomatous_disease_relative	1	0	1	1
local excision or destruction of lesion or tissue of trachea	Local Excision or Destruction of Lesion or Tissue of Trachea	This refers to a surgical procedure that involves removing or destroying abnorm...	True ,False	Local Excision or Destruction of Lesion or Tissue of Trachea	This refers to a surgical procedure where a lesion or tissue in the trachea is e...	Yes,No	local_excision_destruction_lesion_tissue_trachea	1	1	1	1
nlm_hemiplegia_lat_type	Hemiplegia laterality type	Hemiplegia laterality type refers to the side of the body (left or right) that i...	Left:Left.Right:Right	National Library of Medicine Hemiplegia Laterality Type	The term refers to a classification utilized by the National Library of Medicine...	Left Hemisphere, Right Hemisphere, Bilateral Hemispheres	nml_hemiplegia_laterality_type	1	1	0	1
chronic_gout_due_renal_im	Chronic gout due to renal impairment,right hip,with tophus	Chronic gout related to renal dysfunction affecting the right hip, characterized...	0:No—1:Yes	Chronic Gout Due to Renal Impairment	Chronic Gout Due to Renal Impairment is a condition where long-standing, recurri...	Yes,No	chronic_gout_due_renal_impairment	1	1	1	1

Detailed Interoperability Test Results for ADNI-ADSXLIST

The table below displays interoperability results for the ADNI ADSXLIST (Alzheimer's Disease Symptoms Checklist) data tables. The Header column contains the column headers from the table. The Title of Closest Match – GenCDE contains the title associated with a generated CDE that scored above the implicit threshold of the auto fuzziness parameter.

Completeness indicates the percentage of fields containing a non-null value for each column where a correct match was found. Compliance reports the percentage of non-null fields that contain expected value ranges, data types, and/or categorical values. Note that most columns in this table did not comply with our set standards. ADNI uses a binary coding convention where 1=Absent and 2=Present. Our permissible value set allows for 0=False/Absent and 1=True/Present as well as a "True" or "False" string. Using 1 and 2 to represent these values is not typical or recommended in forward-looking data science. The columns that scored 100% compliance were categorized as 'Free Entry'.

This test was performed during the earlier stages of CDE repository development and prior to ingestion of aliases or the generation of new CDEs from ADNI materials.

Supplementary Table 5: Test Result for ADNI-ADSXLIST Interoperability

Header	Title of Closest Match – GenCDE	Completeness (%)	Compliance (%)	Source of GenCDE	ADNI Data Dictionary Definition
Phase	Cortical evoked potential steady-state time	x	x	NLM CDE Repository	ADNI Cohort ID
ID	Participant ID	x	x	CARD CDEs	Record ID
PTID	Participant ID	100	100	CARD CDEs	Participant ID
RID	Healthy Volunteer of PID	x	x	SNOMED CT	Participant roster ID
SITEID	Exostosis of Unspecified Site (ICD9,	x	x	ICD	Site ID
VISCODE	Imaging aneurysm viscous dissipation	x	x	NLM CDE Repository	Visit code
VISCODE2	Scored Below Threshold for Any Potential	x	x	x	Translated visit code
USERDATE	Date of heaviest alcohol use	x	x	PUMAS	Date Record Created
USERDATE2	Date of heaviest alcohol use	x	x	PUMAS	Date Record Last Updated
EXAMDATE	Cutaneous Candidiasis (ICD10, B37.2)	x	x	ICD	Examination Date
AXNAUSEA	Interview History of Nausea	100	0	CTDB Form Legend	Nausea
AXVOMIT	Adverse Drug Reaction Question 16: Black	x	x	CTDB Form Legend	Vomiting
AXDIARRH	Diarrhea	100	0	CTDB Form Legend	Diarrhea
AXCONSTP	Cholecystitis	x	x	BTRIS Clinical	Constipation
AXABDOMN	Abdominal Actinomycotic Infection (ICD10,	x	x	ICD	Abdominal Pain
AXSWEATN	Anhidrosis (ICD10, L74.4)	100	0	ICD	Sweating
AXDIZZY	Becoming dizzy or falling	100	0	PUMAS	Dizziness
AXENERGY	Fatigue (feel tired or heavy, ache)	100	0	Neuropsychiatric	Low Energy
AXDROWSY	Glaucoma drops name PhenX	x	x	NLM CDE Repository	Drowsiness

Supplementary Table 5 (continued)

Header	Title of Closest Match – GenCDE	Completeness (%)	Compliance (%)	Source of GenCDE	ADNI Data Dictionary Definition
AXVISION	Blurred Vision	100	0	BTRIS Clinical	Blurred Vision
AXHDACHE	Headache	100	0	BTRIS Clinical	Headache
AXDRYMTH	Systemic Lupus Eryth	x	x	BTRIS Clinical	Dry Mouth
AXBREATH	Halitosis (ICD10, R19.6)	x	x	ICD	Shortness of Breath
AXCOUGH	Cough (ICD10, R05)	100	0	ICD	Coughing
AXPALPIT	Pale Optic Nerves	x	x	BTRIS Clinical	Palpitations
AXCHEST	Acq Chest Deformity (ICD9, 738.3)	x	x	ICD	Chest Pains
AXURNDIS	Unilat Rad Neck Dissect (ICD9, 40.41)	x	x	ICD	Urinary discomfort (e.g., burning)
AXURNFRQ	Scored Below Threshold for Any Potential	x	x	x	Urinary frequency
AXANKLE	Ankle Swelling	100	0	BTRIS Clinical	Ankle Swelling
AXMUSCLE	Other Plastic Operations on Muscle	x	x	BTRIS Clinical	Musculoskeletal pain
AXRASH	Adverse Drug Reaction Question 1B:	x	x	CTDB Form Legend	Rash
AXINSOMN	Insomnia, Unspecified (ICD10, G47.00)	100	0	ICD	Insomnia
AXDPMOOD	Stroke Specific Quality of Life Scale	x	x	NLM CDE Repository	Depressed mood
AXCRYING	Excessive Crying Of Child, Adolescent, Or	100	0	ICD	Crying
AXELMOOD	Epiretinal membrane in the right eye	x	x	BTRIS Clinical	Elevated Mood
AXWANDER	Water Intake Measurement	x	x	CTDB Form Legend	Wandering
AXFALL	Other and unspecified accidental fall	100	0	BTRIS Clinical	Fall
AXOTHER	OTHER	100	100	BTRIS Clinical	Other/Diagnosis
AXSPECIF	COVID-19 Specific Medication Specify	x	x	NLM CDE Repository	If Other symptoms/diagnosis,
update_stamp	Gastrostomy, Open; without Construction	x	x	CPT-HCPCS	Update stamp
